# Genotype of *PAX2*-related disorders correlates with kidney and ocular manifestations

**DOI:** 10.1038/s41431-025-01822-z

**Published:** 2025-02-24

**Authors:** Ji Hyun Kim, Yo Han Ahn, Yeonji Jang, Eujin Park, Hajeong Lee, Seong Heon Kim, Ji Yeon Song, Kyoung Hee Han, Jiwon Jung, Joo Hoon Lee, Hee Gyung Kang, Jae Ho Jung, Hae Il Cheong

**Affiliations:** 1 Department of Pediatrics, Seoul National University Bundang Hospital, Seongnam, Republic of Korea; 2 Department of Pediatrics, Seoul National University College of Medicine, Seoul, Republic of Korea; 3 Department of Pediatrics, Seoul National University Children’s Hospital, Seoul, Republic of Korea; 4 Kidney Research Institute, Medical Research Center, Seoul National University, Seoul, Republic of Korea; 5 Department of Ophthalmology, Uijeongbu Eulji Medical Center, Eulji University School of Medicine, Uijeongbu, Gyeonggi-do Republic of Korea; 6 Department of Pediatrics, Korea University Guro Hospital, Seoul, Republic of Korea; 7 Department of Internal Medicine, Seoul National University Hospital, Seoul, Republic of Korea; 8 Department of Internal Medicine, Seoul National University College of Medicine, Seoul, Republic of Korea; 9 Department of Pediatrics, Pusan National University Children’s Hospital, Yangsan, Republic of Korea; 10 Department of Pediatrics, Jeju National University, College of Medicine, Jeju, Republic of Korea; 11 Department of Pediatrics, Asan Medical Center Children’s Hospital, University of Ulsan College of Medicine, Seoul, Republic of Korea; 12 Department of Ophthalmology, Seoul National University Children’s Hospital and Seoul National University College of Medicine, Seoul, Republic of Korea; 13 Department of Pediatrics, Seoul Red Cross Hospital, Seoul, Republic of Korea

**Keywords:** Disease genetics, Genetic counselling

## Abstract

*PAX2*-related disorders encompass renal coloboma syndrome (RCS) and hereditary focal segmental glomerulosclerosis (FSGS) type 7. We retrospectively analyzed 27 Korean patients with *PAX2* pathogenic variants detected between 2004 and 2022 and conducted a literature review of 328 cases, including 301 previously reported. In our cohort, 19 had RCS, 4 had FSGS, and 4 had isolated congenital anomalies of the kidneys and urinary tract. Patients were classified by variant type into predicted loss of function (pLoF) and non-pLoF variant groups, and by variant location into paired domain and other sites group. pLoF variants were predominantly associated with RCS, observed in 82% of patients in both our data (18 of 22, *P* = 0.017) and the literature (140 of 171, *P* < 0.001). Kidney failure developed in 52% of Korean patients at a median age of 14.5 years, with no difference in kidney survival between variant types. However, the literature review indicated faster progression to kidney failure in patients with pLoF variants (11.0 vs. 24.0 years; pLoF, *n* = 138 vs. non-pLoF, *n* = 71; *P* = 0.002), with no significant difference by variant location. Ocular manifestations were more common, had earlier onset, and were more severe in the pLoF variants group in our cohort (*P* = 0.038). The literature confirmed a higher prevalence of ocular involvement in patients with pLoF variants (pLoF, *n* = 175 vs. non-pLoF, *n* = 88; *P* < 0.001) and in those with paired domain variants (*P* = 0.01). pLoF variants in *PAX2* were associated with worse kidney and ocular outcomes. These findings support genotype-phenotype correlations, contributing to tailored management in patients with *PAX2*-related disorders.

## Introduction

Renal-coloboma syndrome (RCS, OMIM 120330) is a rare form of chronic kidney disease (CKD) accompanied by retinal coloboma, which was first described by Weaver et al. [1]. It is characterized by renal hypodysplasia (RHD) and optic disc anomalies [2, 3]. In 1995, *PAX2*, paired box gene 2, was found to be the causative gene of RCS, as well as vesicoureteral reflux and renal dysplasia [4]. The *PAX2* gene was the first specific gene identified to be associated with congenital anomalies of the kidneys and urinary tract (CAKUT) [4]. Currently, *PAX2* pathogenic variant is recognized as the second most common genetic cause of CAKUT, with or without optic disc anomalies [5–8]. Moreover, in 2014, *PAX2* was found to be associated with adult-onset focal segmental glomerulosclerosis (FSGS) and listed as a causative pathogenic variant of FSGS (FSGS7, OMIM 616002), a type of glomerulopathy [9–11]. In addition, some reports have demonstrated that *PAX2* pathogenic variants were detected in patients with CKD of unknown etiology [12–14].

*PAX2* is a transcription factor that plays key roles in development of the kidneys, eyes, ears, and urogenital tract. During kidney development, *PAX2* suppresses apoptosis in the developing ureteric bud; therefore, *PAX2* pathogenic variants increase apoptosis during the development of the kidneys and urinary tract, which may underlie the decreased nephron number, hypertrophy of the remaining nephrons, and RHD [15–17]. On the other hand, *PAX2* also suppresses *WT1*, an important transcription factor of podocytes, and its pathogenic variant causes congenital nephrotic syndrome and FSGS. A reduction in *PAX2* expression in the visceral epithelium of the future glomeruli of the S-shaped body is accompanied by a marked increase in *WT1* expression [18], and an animal model demonstrated that *PAX2* re-expression in mature podocytes could be related to glomerular diseases [19]. With the advent of genetics, recent studies have reported variable and overlapping phenotypes of *PAX2*-related disorders [20, 21]. However, limited data have reported no clear genotype–phenotype correlation in the type or location of *PAX2* pathogenic variants regarding kidney outcomes, and longitudinal data on clinical manifestations are lacking. In this study, we investigated the genotype-phenotype correlations of kidney and ocular involvement, including long-term clinical outcomes in patients with *PAX2* pathogenic variants. Moreover, we reviewed all published cases to date of *PAX2*-related disorders to obtain more robust evidence for the genotype-phenotype correlations.

## Materials and methods

### Study participants

This retrospective cohort study was conducted at four medical centers in South Korea. A total of 27 Korean patients with genetically confirmed *PAX2* pathogenic variants between August 2006 and May 2022 were enrolled (Table 1). The diagnosis of *PAX2* was based on Sanger sequencing, targeted exome sequencing, and whole exome sequencing. All the patients were unrelated, except for one sibling (cases 1 and 2). Thirteen cases have been published in previous studies (Supplementary Table S2) [22–26]. Clinical and laboratory data were obtained through a retrospective review of electronic medical records.

**Table 1:** Genotypes and initial presentation of patients with *PAX2* pathogenic variants.

Case	Sex	cDNA	Protein	Onset age (years)	Initial presentation	Age at last visit (years)
*Renal coloboma syndrome*						
1	M	c.76dupG	p.Val26Glyfs*28	7.2	Esotropia	35.5
2	M	c.76dupG	p.Val26Glyfs*28	3.8	Nystagmus	32.4
3	M	c.76dupG	p.Val26Glyfs*28	0.2	Microphthalmia	28.6
4	F	c.76dupG	p.Val26Glyfs*28	6.6	Proteinuria	29.7
5	M	c.76dupG	p.Val26Glyfs*28	0.5	Nystagmus	Death at 9.1
6	M	c.310 C > T	p.Arg104*	0.3	Nystagmus	17.5
7	M	c.754 C > T	p.Arg252*	3	Nystagmus	18.8
8	M	c.76dupG	p.Val26Glyfs*28	0.1	CKD	20.5
9	M	c.344 G > C	p.Arg115Pro	0.1	CKD	24.4
10	M	c.535_546delinsT	p.Asn179Trpfs*17	0.1	Abnormal prenatal USG	5.9
11	M	c.76dupG	p.Val26Glyfs*28	0.1	CKD	13.1
12	F	c.76dupG	p.Val26Glyfs*28	0.1	Abnormal prenatal USG	6.4
13	M	c.343 C > T	p.Arg115*	0.1	Abnormal prenatal USG	11.4
14	F	c.860delA	p.Gln287Argfs*10	10.1	Proteinuria	10.9
15	M	c.754 C > T	p.Arg252*	0.3	Nystagmus	13
16	M	c.76dupG	p.Val26Glyfs*28	0.1	Abnormal prenatal USG	5.4
17	F	c.76delG	p.Val26Cysfs*3	0.1	Abnormal prenatal USG	16.4
18	F	c.76dupG	p.Val26Glyfs*28	10.2	CKD	43.1
19	M	c.361_373dup	p.Asp125Glyfs*5	4.5	Proteinuria	33
*Focal segmental glomerulosclerosis*						
20	M	c.76dupG	p.Val26Glyfs*28	5.2	Proteinuria	29.4
21	M	c.223_226dup	p.Gly76Aspfs*27	13.4	Proteinuria	27.5
22	F	c.74 G > A	p.Gly25Glu	7.3	Proteinuria	23.8
23	M	c.419 G > A	p.Arg140Gln	7.8	Proteinuria	11.6
*Isolated congenital anomalies of the kidney and urinary tract*						
24	M	c.686-1 G > T	-	9.1	Proteinuria	12.9
25	F	c.832 C > T	p.Gln278*	6.1	Proteinuria	11.6
26	F	c.1052 G > T	p.Gly351Val	0.1	Proteinuria	1.6
27	M	c.206 T > C	p.Leu69Pro	9.1	Proteinuria	31.2

The reference sequence of PAX2 gene is NM_003990.5.

*M* male, *F* female, *PU* proteinuria, *CKD* chronic kidney disease, *USG* ultrasonography.

### Genotype-phenotype correlation

In our Korean cohort, the clinical phenotypes were categorized as RCS, FSGS, or isolated CAKUT. The diagnosis of CAKUT was made based on kidney imaging studies. Additionally, RHD was defined as abnormally small kidneys with poor corticomedullary differentiation and increased renal parenchymal echogenicity upon kidney imaging, whereas RCS was defined as CAKUT with ocular involvement. Furthermore, FSGS was diagnosed based on histopathological findings of kidney biopsy, characterized by sclerosis affecting some glomeruli and involving only segments of the affected glomeruli. We categorized cases as FSGS in the absence of kidney morphological abnormalities, regardless of ocular findings. The estimated glomerular filtration rate (eGFR) was calculated using the bedside Schwartz equation for patients under 18 years of age and the CKD Epidemiology Collaboration 2021 formula for patients aged 18 years and older [27, 28]. CKD was defined as an eGFR less than 90 mL/min per 1.73 m^2^ and stages of CKD (G2–4) are defined by the KDIGO guidelines. Ocular involvement with *PAX2* variants was defined as optic disc dysplasia (abnormal development of the optic disc, such as optic disc excavation or hypoplasia) with numerous cilioretinal vessels [29]. Fundus photographs were obtained from all patients, and an experienced ophthalmologist reviewed photographs of the fundus of both eyes of each patient. A Snellen chart was used to measure the best corrected visual acuity, which was converted to the logarithm of the minimum angle of resolution (logMAR) value for analysis. Favorable visual acuity was defined as a best visual acuity of 20/40 or more at nadir. Furthermore, visual field testing was performed using Goldmann or Humphrey visual perimetry, and cross-sectional images of the optic disc and macular area were evaluated using spectral domain optical coherence tomography (Cirrus HD-OCT, Carl Zeiss Meditec, Dublin, CA, USA) when further examination was required.

To evaluate the genotype-phenotype correlation, the patients were grouped into the pLoF variant group and non-pLoF variant group according to variant type in *PAX2*. Pathogenic nonsense, frameshifting, splice site variants, and large deletion were classified as the pLoF variants, whereas missense and in-frame variants were classified as the non-pLoF variants. For analysis based on the location of the variant, the non-pLoF variant group was further grouped into the paired domain group and other sites group in *PAX2*. Additionally, we evaluated kidney and ocular outcomes according to variant type and location in the *PAX2* gene.

### Literature review for PAX2‑related disorder

To elucidate the genotype-phenotype correlations of *PAX2*-related disorders involving kidney and ocular manifestations by variant type and location, we conducted a comprehensive review of all published cases to date. The literature search was conducted in PubMed using terms such as “*PAX2*,” “renal coloboma syndrome,” “papillorenal syndrome,” “FSGS,” “CAKUT,” “genotype,” and “phenotype”. This research included original articles, reviews, case reports, and abstracts, and was performed in the last week of November 2024. Data on ophthalmic and kidney phenotypes were collected. Cases with unclear or incomplete information on kidney outcomes, such as the age at the onset of kidney failure and kidney function status were excluded from the survival analysis. For ophthalmic phenotypes, only the presence of involvement was analyzed due to the limited availability of detailed data. Phenotypic data were obtained from the original publications.

### Statistical analysis

SPSS version 27.0 (Armonk, NY, USA) was used for statistical analysis. Values are presented as median and interquartile range (IQR). Categorical variables were analyzed using Chi-square test or Fisher’s exact test and continuous variables were compared using the Mann–Whitney U test or Kruskal–Wallis test. The effect on long-term kidney survival rates (age at kidney failure) and the onset of ocular manifestations was analyzed using Kaplan–Meier survival probability estimates and a log-rank test. The hazard ratio (HR) was analyzed using the Cox proportional hazards model. A value of *P* < 0.05 was considered statistically significant.

## Results

In our Korean cohort (*n* = 27), 19 patients were diagnosed with RCS, 4 with FSGS, and 4 with isolated CAKUT without ocular manifestations. The median onset age and age at the last follow-up were 1.8 (IQR, 0.1–7.0) and 17.0 (IQR, 10.5–27.8) years, respectively (Table 1). The most common initial presentation was proteinuria (*n* = 10), followed by ocular symptoms such as nystagmus and microphthalmia (*n* = 7), abnormalities on prenatal kidney ultrasonography (USG) (*n* = 5), and CKD (*n* = 5). The median age at genetic diagnosis was 12.4 (IQR, 8.8–19.0) years.

### Genetic diagnosis in our Korean cohort

**Fig. 1:**
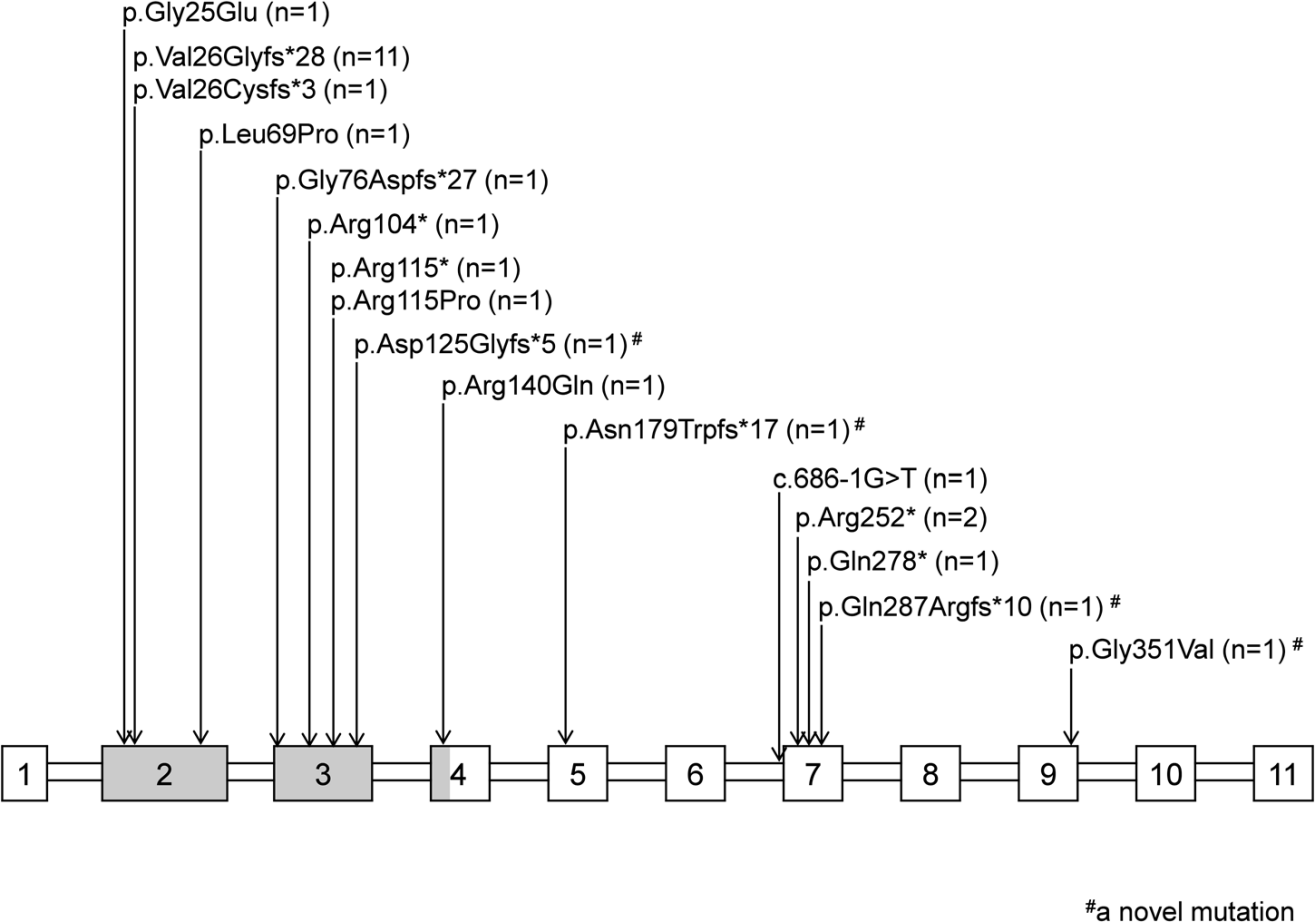
Pathogenic variants in the *PAX2* gene identified in our study. The gray colored boxes show the paired domain of the PAX2 protein. # a novel mutation.

*PAX2* gene variants were identified by Sanger sequencing in eight patients with characteristic RCS. Targeted exome sequencing using a disease-causing gene panel for CAKUT, steroid-resistant nephrotic syndrome, or cystic kidney disease, was conducted in twelve patients based on their clinical manifestations [22–24]. Whole exome sequencing detected *PAX2* variants in seven patients for CKD of unknown etiology. A total of fourteen different *PAX2* pathogenic variants were identified, including 10 known and 4 novel pathogenic variants (Fig. 1). Parental screening for pathogenic variants was performed in 13 patients. Among these, three cases (cases 15, 24, and 26) inherited their *PAX2* pathogenic variants from one parent, who presented with kidney and/or ocular manifestations. Eight patients were confirmed to have de novo pathogenic variants. In the remaining two familial cases (cases 1 and 2), no pathogenic variants were detected in the parents, suggesting the presence of germline mosaicism [25]. Additionally, none of the 14 patients without family screening had a positive family history of *PAX2* pathogenic variant-related phenotypes.

### Kidney manifestations in our Korean cohort

In the 27 patients, the median age at the onset of kidney manifestations was 6.1 (IQR, 0.1–9.1) years (Table 2). At the time of kidney problem diagnosis, 22 (81.5%) of the 27 patients already had CKD, and 11 were infants. Fourteen (53.8%) patients developed kidney failure at a median age of 14.5 (95% confidence interval (CI), 11.9–17.1; range, 0.2–17.5) years, which was estimated by Kaplan–Meier analysis.

**Table 2:** Kidney and ocular manifestations in patients with *PAX2* pathogenic variants.

Case	Kidney symptoms and age at onset (symptoms (years))	USG and biopsy findings (USG; biopsy)	Last kidney outcome (years)	Ocular symptoms and age at diagnosis (symptoms (years))	Optic disc (R/L)	Central retinal artery and Number of cilioretinal artery^a^ (R/L)	Visual acuity (R;L)	Others (R/L)
*Renal coloboma syndrome*								
1	PU (12)	RHD; FSGS	KFRT (12.2)	Strabismus (7.2)	E + PPA + VS/E + PPA	Abs, 8/Abs, 7	20/20;20/200	-/RD
2	PU (8)	RHD, horseshoe kidney; ND	KFRT (17.5)	Nystagmus (3.8)	E + PPA/E + PPA	Abs, 8/Abs, 7	20/63;20/320^b^	-/RD
3	PU, CKD (9.7)	RHD; FSGS	KFRT (14.8)	Microphthalmia (0.2)	E + PPA/E + PPA	Partial, 6/Abs, 8	20/20;CF	-/CRA
4	PU (6.6)	RHD; ND	KFRT (8.6)	Work up (7.0)	E + PPA/E + PPA	Abs, 9/Abs, 8	20/16;20/16	VFD/VFD
5	PU, CKD (1)	RHD; ND	KFRT (7.1)	Nystagmus (0.5)	E + PPA/E + PPA	Abs, 6/Abs, 5	UC^c^;UC	CRA/CRA
6	PU, CKD (1)	RHD, CC; ND	KFRT (9.5)	Nystagmus (0.3)	Rudimentary OD/E + PPA	Abs, 8/Abs, 9	20/200;20/25	CRA/-
7	PU, CKD (15)	RHD; ND	CKD G3 (18.8)	Nystagmus (3.0)	Rudimentary OD/E + PPA	Abs, 8/Abs, 10	UC^c^;UC	CRA/CRA
8	CKD (0.1)	RHD, CC; ND	KFRT (3.2)	Work up (9.1)	E + PPA/E + PPA	Partial, 11/Partial, 8	20/20;20/20	VFD/ VFD
9	CKD (0.1)	RHD, CC; ND	KFRT (10.4)	Work up (19.4)	E + PPA/E + PPA	Abs, 11/Abs, 8	20/20;20/20	(-/-)
10	Abn pUSG (0.1)	RHD, CC; ND	CKD G2 (5.9)	Work up (0.9)	E + PPA/E + PPA	Abs, 10/Abs, 8	UA;UA	CRA/-
11	CKD (0.1)	RHD, CC; ND	CKD G4 (13.1)	Work up (11.0)	E + PPA/E + PPA	Abs, 11/Abs, 12	20/16;20/32	(-/RS^d^)
12	Abn pUSG (0.1)	RHD, CC; ND	CKD G4 (6.4)	Work up (5.3)	E/E	Abs, 14/Abs, 10	20/20;20/20	(-/-)
13	Abn pUSG (0.1)	RHD, CC; ND	CKD G3a (11.4)	Work up (10.7)	E + PPA/E+Seg hypo	Abs, 11/Abs, 10	20/80;20/40	-/RS
14	PU (10.1)	RHD; ND	CKD G3a (10.9)	Work up (10.7)	Normal/E + PPA+Peripapillary RS + RS	Cplt, 9/Abs, 17	20/20;20/32	RS/RS
15	Abnormal USG (8.5)	RHD, CC; ND	CKD G2 (13.0)	Nystagmus, Microphthalmia (0.3)	PHPV/Rudimentary OD	Abs, UC/Abs, UC^e^	LP (-);LP (-)	PHPV /Retina aplasia
16	Abn pUSG (0.1)	RHD, CC; ND	CKD G3a (5.4)	Work up (5.4)	E + PPA/E + PPA	Abs, 14/Partial, 11	20/30;20/30	(-/-)
17	Abn pUSG (0.1)	RHD, CC; ND	KFRT (0.2)	Work up (16.3)	Hypo/Hypo+PPA	Cplt, 3/Abs, 8	20/20;20/20	(-/-)
18	CKD (10.2)	RHD, CC, hydronephrosis; FSGS	KFRT (12.9)	Decreased visual acuity (30.2)	E+HypoVS+PPA/E + VS + PPA	Abs, 11/Abs, 12	20/125;20/20	RS/-
19	PU (4.5)	RHD and left vesicoureteral reflux; ND	KFRT (8.9)	Work up (29.4)	E + PPA/E + PPA	Abs, 10/Abs, 11	20/20;20/20	Corneal dystrophy
*Focal segmental glomerulosclerosis*								
20	PU (5.2)	No anomaly; FSGS	KFRT (15.5)	Work up (19.3)	Seg hypo/E + PPA	Partial, 12/Partial, 9	20/16;20/20	VFD/ VFD
21	PU (13.4)	No anomaly; FSGS	CKD G3b (27.5)	Work up (22.9)	Seg hypo+Peripapillary RS + VS/Seg hypo /Seg hypo	Cplt, 6/Cplt, 8	20/25;20/20	(-/-)
22	PU (7.3)	No anomaly; FSGS	KFRT (14.5)	Work up (21.4)	Normal /Seg hypo+PPA	Cplt, 2/Cplt, 7	20/20;20/20	(-/-)
23	PU (7.8)	No anomaly; FSGS	CKD G2 (11.6)	Work up (10.1)	Normal/Normal	Partial, 6/Partial, 8	20/20;20/20	(-/-)
*Isolated congenital anomalies of the kidney and urinary tract*								
24	PU (9.1)	RHD; ND	CKD G2 (12.9)	Work up (11.0)	Normal/Normal	Abs, 8/Abs, 7	20/16;20/16	(-/-)
25	PU (6.1)	RHD; ND	CKD G3b (11.6)	Work up (7.5)	Normal/Seg hypo	Cplt, 4/Cplt, 6	20/20;20/20	(-/-)
26	CKD (0.1)	RHD, CC; ND	CKD G3b (1.6)	Work up (0.1)	Normal/Normal	Cplt, UA/Cplt, UA	UA;UA	(-/-)
27	PU (9.1)	RHD, CC; ND	KFRT (9.7)	Work up (30.9)	Normal/Normal	Partial, 9/Cplt, 5	20/20;20/20	(-/-)

*USG* ultrasonography, *CKD* chronic kidney disease, *KFRT* kidney failure with replacement therapy, *PU* proteinuria, *Abn* *pUSG* abnormal prenatal ultrasonography, *RHD* renal hypodysplasia, *CC* cortical cysts, *FSGS* focal segmental glomerulosclerosis, *ND* not done, *R* right, *L* left, *E* Optic disc excavation, *PPA* peripapillary atrophy, *VS* vitreous strand on optic disc, *RD* retinal detachment, *Abs* absence, *Cplt* complete, *CF* counting fingers, *CRA* chorioretinal atrophy, *VFD* visual field defect, *UC* uncheckable, *OD* Optic disc, *RS* retinoschisis, *Hypo* hypoplasia, *Seg hypo* segmental hypoplasia, *LP* light perception, *PHPV* persistent hyperplastic primary vitreous, *UA* unavailable data.

^a^Based on the maximum number of cilioreretinal vessels in both eyes.

^b^Underwent retinal surgery due to rhegmatogenous retinal detachment.

^c^Un-checkable due to development delay.

^d^Spontaneously restored retinal structure.

^e^Un-checkable due to severe retinal anomalies.

Twenty-three patients with RCS and isolated CAKUT were diagnosed with hypodysplastic kidneys, cortical cysts, hydronephrosis, or horseshoe kidney on USG for the following reasons: proteinuria and/or CKD found incidentally during school screening or routine laboratory findings during hospitalization (*n* = 17), and abnormalities on USG through prenatal USG (*n* = 5) or incidental USG (*n* = 1). Proteinuria was found at the time of diagnosis of kidney manifestations in all RCS and isolated CAKUT patients, except in case 15, whose eGFR was 87 mL/min/m^2^ at the last follow-up of 13.0 years. Kidney biopsy, conducted in three patients (cases 1, 3, and 18) at a median age of 10.2 (IQR, 10.0–12.6) years, revealed findings compatible with FSGS. All RCS and isolated CAKUT patients developed CKD during childhood at a median age of 4.5 (IQR, 0.1–9.4) years, and 11 of them required kidney replacement therapy at a median age of 15.0 (IQR, 14.5–15.5) years.

Four patients with FSGS initially presented as incidentally found asymptomatic proteinuria at school screening or routine laboratory screening at the median age of 7.6 (IQR, 6.3–10.6) years. At the time of FSGS diagnosis, there were no abnormalities on kidney USG. Additionally, proteinuria did not respond to corticosteroids and/or calcineurin inhibitors. Kidney biopsy revealed 5.9–62.5% sclerotic glomerular lesions with tubular atrophy and interstitial fibrosis at a median age of 8.7 (IQR, 6.8–10.9).

### Ocular manifestations in our Korean cohort

Visual acuity ranged from 20/20 to no light perception (Table 2). Forty-one eyes (70.7%) had ≥20/40 visual acuity. Ophthalmic abnormalities, such as congenital nystagmus, congenital strabismus, microphthalmia, and amblyopia, were the first manifestations of *PAX2*-related disease in seven patients, while ocular involvement in the other patients was identified through a workup after the diagnosis of kidney problems.

**Fig. 2:**
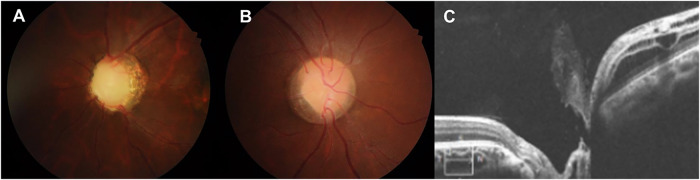
Optic disc abnormalities in patients with *PAX2* pathogenic variants. **A** Excavated optic disc with numerous cilioretinal vessels and absence of a central retinal vessel in case 1; **B** Optic disc segmental hypoplasia and numerous cilioretinal vessels in case 20; **C** Optical coherence tomography image of peripapillary retinoschisis and vitreous strands on the optic disc in case 21.

**Fig. 3:**
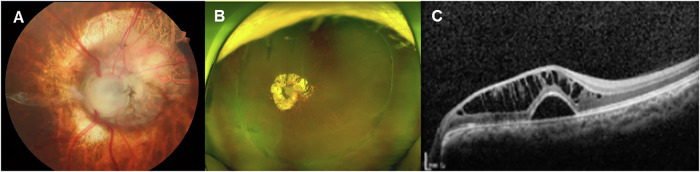
Retinal abnormalities in patients with *PAX2* pathogenic variants. **A** Severe chorioretinal atrophy with optic disc dysplasia in case 6; **B** Post-encircling surgery state due to retinal detachment in case 2; **C** Optical coherence tomography image of retinoschisis and subfoveal retinal detachment in case 11.

Four (14.8%) patients had normal ocular findings, while nineteen (70.4%) patients with *PAX2* pathogenic variants had optic disc excavation with numerous cilioretinal vessels and absence of central retinal vessels, which are the most typical ocular findings in *PAX2*-related disorders (Fig. 2A). Although these findings were the most common optic disc abnormality observed in *PAX2*-related disorders, various optic disc dysplasias, such as hypoplastic optic disc with peripapillary atrophy, optic disc segmental hypoplasia (Fig. 2B), and peripapillary retinoschisis and vitreous strand (Fig. 2C) were also identified. Most patients with optic disc dysplasia also demonstrated anomalous retinal vasculature, numerous cilioretinal vessels, and rudimentary central retinal vessels. Interestingly, twenty-five eyes had additional ocular abnormalities combined with optic disc dysplasia as follows: diffuse chorioretinal atrophy (Fig. 3A, 7 eyes), retinal detachment (Fig. 3B, 2 eyes), microphthalmia (1 eye), retinal agenesis (1 eye), persistent hyperplastic primary vitreous (1 eye), retinoschisis on macular (Fig. 3C, 3 eyes) or on peripapillary area (Fig. 2C, 2 eyes), visual field defect (6 eyes), and corneal dystrophy (2 eyes).

### Genotype-phenotype correlation in our Korean cohort

The c.76dupG pathogenic variant, which has been identified as a mutational hotspot for *PAX2* in the Leiden Open Variation Database (http://www.lovd.nl/3.0/home), was the most common in 11 (40.7%) patients: 10 with RCS and 1 with FSGS. Twenty-two (81.5%) patients harbored pLoF variants, while five (18.5%) had non-pLoF variants, of which four were located in the paired domain of *PAX2*. There were no statistical differences in sex, age at onset, initial presentation, age at kidney manifestations, and developmental delay between the pLoF variant group (*n* = 22) and the non-pLoF variant group (*n* = 5) (Table 3). RCS was significantly more prevalent among patients with pLoF variants, occurring in 18 (81.8%) of these patients, compared to one (20%) in the non-pLoF variant group (*P* = 0.017). Kidney survival did not differ between the pLoF and non-pLoF variant groups (*P* = 0.403).

**Table 3:** Comparison of patient phenotypes according to the genotype of *PAX2* pathogenic variants.

	Predicted loss of function (*n* = 22)	Non-Predicted loss of function (*n* = 5)	*P* value
Sex, male: female	16:06	3:02	0.616
Age at onset, years	1.8 (0.1–6.6)	7.0 (0.1–7.8)	0.726
Age at genetic diagnosis, years	12.4 (8.8–16.5)	19.0 (10.9–23.1)	0.492
Age at the last follow up, years	17.0 (11.4–29.4)	23.8 (11.6–24.4)	0.876
*Initial manifestations*			0.283
Ocular symptoms	7 (31.8)	0	
Kidney symptoms	15 (68.2)	5 (100.0)	
*Clinical phenotype*			0.034
Renal coloboma syndrome	18 (81.8)	1 (20.0)	
Focal segmental glomerulosclerosis	2 (9.1)	2 (40.0)	
Isolated CAKUT	2 (9.1)	2 (40.0)	
Developmental delay	4 (18.2)	0	0.561
*Kidney manifestations*			
Age at kidney manifestations, years	5.7 (0.1–9.7)	7.0 (0.1–7.8)	0.679
Proteinuria at initial diagnosis	21 (95.5)	5 (100.0)	1
CKD at initial diagnosis	18 (81.8)	4 (80.0)	1
Median age at the onset of CKD, years (95% CI)^a^	6.6 (0.0–14.5)	5.1 (0.0–15.8)	0.274
Median age at kidney failure, years (95% CI)^a^	14.8 (10.1–19.5)	10.4 (7.3–13.5)	0.403
*Ocular manifestations*^b^	44 eyes	10 eyes	
Optic disc anomaly			<0.001
Anomalous optic disc	42 (95.5)	1 (10.0)	
Normal optic disc	2 (4.5)	9 (90.0)	
Central retinal artery			0.018
Absence	30 (68.2)	2 (20.0)	
Partial	6 (13.6)	3 (30.0)	
Complete	8 (18.2)	5 (50.0)	
Number of cilioretinal vessel	8.0 (10–7.5)^c^	7.5 (8.0–6.2)	0.048
Presence of retinopathy	16 (38.1)	0 (0.0)	0.011
Visual acuity^d^, mean (maximum-minimum)	20/22.5 (20/20–light perception)	20/20 (20/20–20/30)	0.041
Visual outcome ≥ 20/40^d^	31/42 (72.5)	10/10 (100)	0.096
Median age at the diagnosis of ocular involvement, years (95% CI)^a^	7.2 (2.3–12.1)	21.4 (18.2–24.6)	0.038

Values are expressed as numbers (%) and medians (interquartile range), except for visual acuity.

*CAKUT* congenital anomalies of the kidney and urinary tract, *CKD* chronic kidney disease, *CI* confidence interval.

^a^It is estimated by Kaplan–Meier analysis.

^b^Ocular manifestations were analyzed separately in both eyes of patients.

^c^Cilioretinal vessels could not be counted in case 15 due to severe retinal anomalies.

^d^Snellen visual acuity was observed in 42 and 10 eyes in the pLoF and missense groups, respectively.

**Fig. 4:**
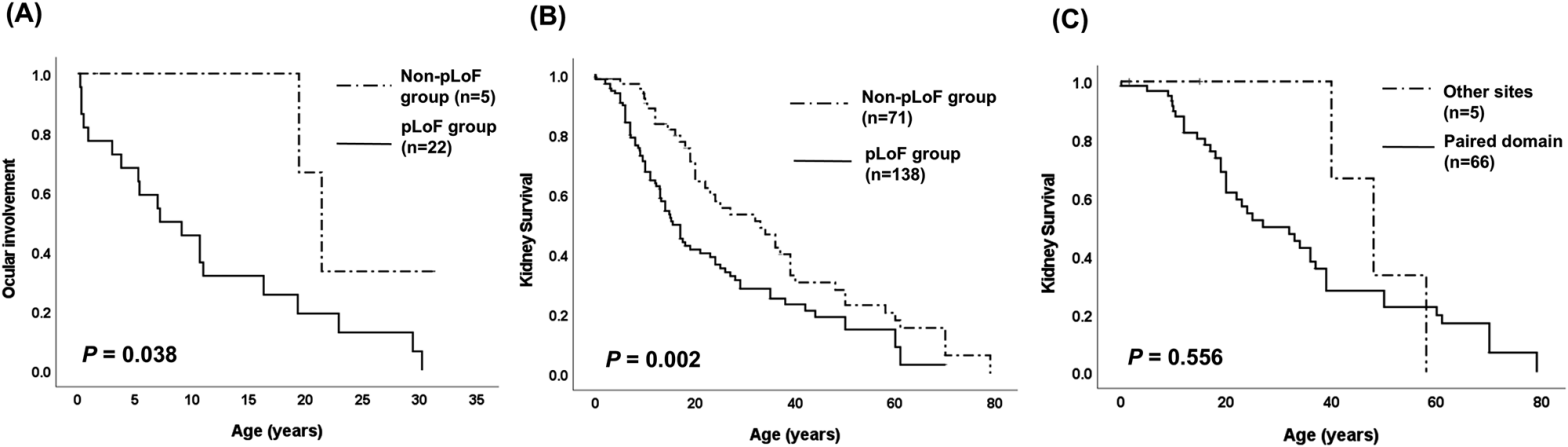
Survival analysis of ocular and kidney outcomes in patients with *PAX2* pathogenic variants. **A** Ocular involvement-free survival analysis in our cohort by *PAX2* pathogenic variant type (*n* = 27). Kidney survival analysis in 209 reported cases with pathogenic *PAX2* variants: **B** Survival analysis for kidney failure by variant type; **C** Survival analysis for kidney failure by variant location.

Severe optic disc anomaly, absence of a central retinal vasculature, coexisting retinopathy, and lower visual acuity were significantly more common in the pLoF variant group compared to the non-pLoF variant group (Table 3). Furthermore, the pLoF variant group was diagnosed with ocular manifestations significantly earlier than the non-pLoF variant group (Fig. 4A, log-rank test, *P* = 0.038).

Due to the small sample size, correlations between the kidney and ocular phenotypes and the location of the variants were not analyzed.

### Literature review of genotype-phenotype correlation in PAX2‑related disorders

A total of 49 articles were included, comprising 24 original articles, 2 reviews, 22 case reports, and 1 abstract, with detailed information on 301 patients provided in Supplementary Table S3. Among 255 patients with analyzable phenotypes including RCS, CAKUT, or FSGS/nephrosis, 140 of 171 (81.9%) patients with pLoF variants presented with RCS, compared to 44 of 84 (52.4%) patients with non-pLoF variants (*P* < 0.001).

For kidney outcome analysis, 209 patients were included: 182 from 37 published papers and 27 from our study. Among these, 138 (66.0%) were classified into the pLoF variant group (95 frameshift, 32 nonsense, 10 abnormal splicing, and 1 large deletion), and 71 (34.0%) were in the non-pLoF variant group (54 missense and 17 in-frame variants). The pLoF variant group developed kidney failure at a significantly earlier median age of 11.0 years (95% CI, 8.6–13.4; range, 0.0–61.0) compared to 24.0 years (95% CI, 11.0–37.0; range, 0.1–79.0) in the non-pLoF variant group (*P* = 0.002, HR 1.8 [95% CI, 1.2–2.6]) (Fig. 4B). Within the non-pLoF variant group, analysis by variant location revealed no difference in kidney outcome between the paired domain group (*n* = 66) and the other sites group (*n* = 5) (*P* = 0.556) (Fig. 4C).

For ocular involvement, 263 patients from 47 articles were analyzed: 175 (66.5%) were classified into the pLoF variant group and 88 (33.5%) in the non-pLoF variant group. Ocular involvement was significantly more prevalent in the pLoF group (142 of 175, 81.1%) compared to the non-pLoF group (45 of 88, 51.1%) (*P* < 0.001, odds ratio (OR) 4.1 [95% CI, 2.3–7.2]). Further analysis of the non-pLoF variant group revealed that ocular involvement was significantly more common in the paired domain group (43 of 76, 56.6%) compared to the other sites group (2 of 12, 16.7%) (*P* = 0.01, OR 6.5 [95% CI, 1.3–31.8]).

## Discussion

To our knowledge, this is the first study to reveal differences in kidney and ocular manifestations and long-term clinical outcomes according to variant types and locations, with a special emphasis on kidney prognosis and ocular involvement. Additionally, we comprehensively identified the genotype-phenotype correlations specifically in the field of nephrology and ophthalmology, incorporating all published cases of *PAX2*-related disorders to date. This approach enhances the understanding of the phenotypic spectrum delineation according to genotypes compared to prior research [20, 21, 30]_._

The PAX2 transcription factor plays a critical role in optic nerve differentiation during eye development. Since Savell and Cook’s report on “isolated colobomas” [31], some terminologies of this disease have been confused. Parsa et al. later clarified that many individuals in the originally reported family likely had optic disc excavation rather than optic disc coloboma, proposing the term ‘papillorenal syndrome’ [29, 32]. Our study demonstrated that optic disc excavation with numerous cilioretinal vessels was the most common finding of *PAX2*-related disorders. In addition, these patients can also have various ocular abnormalities beyond optic disc anomalies, such as retinal detachment, retinoschisis, chorioretinal atrophy, corneal dystrophy, and microphthalmia. However, these terms remain inadequate, as there is no evidence of optic disc coloboma and the ocular abnormalities extend beyond the optic disc [29]. In addition, kidney manifestations are diverse, including CAKUT, FSGS, and cystic kidney disease, as well as other extrarenal manifestations such as developmental delay, skeletal deformity, and congenital heart abnormalities [20, 21]_._ Therefore, as suggested in previous studies, we advocate for the adoption of ‘*PAX2*-related disorder’ as a more inclusive terminology for renal-ocular abnormalities related to *PAX2* variants [20, 21, 30].

Consistent with previous studies, our findings in the Korean cohort demonstrated phenotypic variability in *PAX2*-related disorders, ranging from typical RCS to FSGS without kidney morphological abnormalities [11, 21, 33–35]. The onset of kidney failure in our cohort varied widely, ranging from 0.2 to 17.5 years, which aligns with the broader range reported in the literature (birth to 79 years) [36, 37].

Regarding genotype-phenotype correlation in terms of the type of variants, the pLoF group exhibited a much higher prevalence of RCS in our Korean cohort and comprehensive review of *PAX2*-related literature to date, consistent with a recent report from China [30], despite the lack of consistent correlation in other reports [20, 21, 37]. Through extensive literature reviews, we found that the timing of kidney failure was significantly associated with the type of pathogenic variants in the *PAX2* gene, with pLoF variants showing a worse kidney prognosis, although no genotype-phenotype correlation was observed in our cohort or other previous studies to date [21, 25, 37]. Regarding variant location, no genotype-phenotype correlation was observed in literature reviews, consistent with previous studies [20, 21].

Additionally, to date, genetic analysis of *PAX2*-related disorders has not shown a relationship between pathogenic variant types and ocular involvement [20, 37]. However, in our Korean study, we observed an association between the severity of ocular abnormalities, visual acuity, and the genotype of the *PAX2* variants. Patients with the pLoF variants in the *PAX2* gene were diagnosed with ocular involvement significantly earlier and had worse visual outcomes compared to those with non-pLoF variants. Extensive literature reviews also showed a higher prevalence of ocular involvement in the pLoF group (*P* < 0.001, OR 4.1). Comprehensive analyses have identified a clear genotype-phenotype correlation in both nephrology and ophthalmology. Consistent with previous reports, optic nerve abnormalities were observed in our Korean cohort patients with FSGS caused by the pLoF variants, but not in those with non-pLoF variants [9–11]. In comparison to previous studies with limited ophthalmic evaluation [20, 21, 30, 37], we conducted detailed ophthalmologic exams in all patients, reviewed by an experienced ophthalmologist. This disparity in evaluation methods could contribute to differences in study results. Nevertheless, the number of non-pLoF variants in our data is small, limiting the interpretation of the association.

Significantly severe nephrologic and ocular phenotypes were observed in *PAX2* patients with pLoF, where haploinsufficiency has been classically understood as the primary disease-causing mechanism of action in these patients. In contrast, non-pLoF variants may mimic these effects but result in relatively milder phenotypes, possibly by reducing *PAX2* transactivation and protein stability without affecting nuclear localization, steady-state mRNA levels, or the ability to bind the DNA consensus sequence [30, 37–39]. However, recent studies have also highlighted the roles of environmental and epigenetic factors, modifying genes, and digenic mechanisms in contributing to the phenotypic variability observed in *PAX2*-related disorders [9, 30, 35, 40].

In our Korean cohort, 24/27 (88.9%) patients with *PAX2* pathogenic variants had optic disc abnormalities, consistent with a previous report [37]. Seven presented with eye-related symptoms, while others had subtle changes detected only on detailed funduscopic examination. Retinal complications also occurred in some cases during follow-up, highlighting the importance of periodic eye examinations to prevent further vision impairment. In addition, typical ocular manifestations could provide useful clues for differential diagnosis in pediatric patients with steroid-resistant nephrotic syndrome/FSGS or CAKUT [41, 42]. In this study, as in previous studies, some patients underwent detailed fundus examination after identifying genetic abnormalities [43, 44]. Recently, genetic screening using targeted exome sequencing or whole exome sequencing has become widely used in clinical practice. It is possible to refine phenotypes based on genetic marker data, which is called reverse phenotyping [45, 46]. This approach can provide the correct diagnosis and motivate surveillance for previously unrecognized clinical manifestations and potential future complications. In addition, *PAX2* pathogenic variants were detected in recent studies that performed genetic diagnoses of patients with CKD of unknown origin [12, 14]_._

*PAX2* pathogenic variants could result in both glomerulopathy and congenital kidney anomalies [44]_._ In our cohort, all patients with RCS and isolated RHD, except one (case 15), had proteinuria at the time their sonographic abnormalities were identified. Kidney biopsy in three patients with RCS revealed glomerulosclerosis, consistent with previous studies of FSGS in some RCS cases [10, 20]. Moreover, Deng et al. revealed that all patients with *PAX2* pathogenic variants had both RHD and proteinuria, with 6/10 having nephrotic-range proteinuria [20]. These findings suggest that CAKUT caused by *PAX2* pathogenic variants might be associated with glomerulosclerosis, contributing to proteinuria and facilitating CKD progression. A genetic analysis in CAKUT patients showed that patients with *PAX2* pathogenic variants developed kidney failure more rapidly than those with other gene pathogenic variants [7]. In this study, most patients with RCS and isolated CAKUT had CKD at initial renal presentation and progressed to kidney failure more rapidly compared to general CAKUT patients, for which a previous study reported a median of 31 years [47]. It could be associated with proteinuria, which is an independent risk factor for CKD progression in patients with non-glomerulopathy [48]. Therefore, patients with CAKUT and proteinuria need to check for *PAX2* pathogenic variants and undergo ophthalmologic examinations, especially when accompanied by childhood-onset CKD.

However, our study had several limitations. First, the small study population limited the generalizability despite efforts to address this through an extensive literature review. Many cases from the included studies lacked detailed and accurate kidney outcome data. For ophthalmic phenotypes, most cases only provided information on the presence or absence of ocular involvement, precluding detailed analyses of specific ocular manifestations and survival outcomes. Furthermore, most identified variants were located in the paired domain, with only five involving variants in other regions. Consequently, genotype-phenotype correlation analyses based on variant location may be limited, and the findings should not be overgeneralized. Second, trio samples were not available for all patients; therefore, the assessment of penetrance or segregation was insufficient. Nonetheless, to our knowledge, this is the first report to perform a genotype-phenotype analysis in patients with *PAX2* pathogenic variants, evaluate long-term clinical outcomes in this cohort, and include an extensive literature review. In addition, detailed ophthalmological problems were found in patients with *PAX2* pathogenic variants, which explains the development of ophthalmic abnormalities. Further studies are needed to achieve a clearer understanding of the phenotypic variations observed in our study.

In conclusion, the clinical phenotypes of *PAX2* pathogenic variants, including RCS, FSGS, and isolated CAKUT, were highly variable. A clear genotype-phenotype correlation was observed in both kidney and ocular manifestations. The pLoF variants were predominantly associated with RCS and exhibited a worse kidney prognosis compared to the non-pLoF variants in literature reviews, although this was not significant in our study. The kidney survival did not differ by variant location. The pLoF variants were also associated with more common occurrence, earlier onset, and severe ocular involvement, which was not confined to the optic disc. The ocular involvement was more common in paired domain variants than in other sites. These insights demonstrate the significant implications of the genotype-based analysis approach in facilitating targeted and expedited patient care.

## Supplementary information

SUPPLEMENTAL MATERIAL

## Data Availability

All data that support the findings of this study are included within this article and its additional supplementary information documents. Additional data are available from the corresponding author upon reasonable request. Data are not publicly available due to privacy concerns.

## References

[1] Weaver RG, Cashwell LF, Lorentz W, Whiteman D, Geisinger KR, Ball M, et al. Optic nerve coloboma associated with renal disease. Am J Med Genet. 1988;29:597–605. 3377002 10.1002/ajmg.1320290318

[2] Nishimoto K, Iijima K, Shirakawa T, Kitagawa K, Satomura K, Nakamura H, et al. PAX2 gene mutation in a family with isolated renal hypoplasia. J Am Soc Nephrol. 2001;12:1769–72. 11461952 10.1681/ASN.V1281769

[3] Benetti E, Artifoni L, Salviati L, Pinello L, Perrotta S, Zuffardi O, et al. Renal hypoplasia without optic coloboma associated with PAX2 gene deletion. Nephrol Dialysis Transpl. 2007;22:2076–8. 10.1093/ndt/gfm187 17403695

[4] Sanyanusin P, Schimmenti LA, McNoe LA, Ward TA, Pierpont MEM, Sullivan MJ, et al. Mutation of the PAX2 gene in a family with optic nerve colobomas, renal anomalies and vesicoureteral reflux. Nat Genet. 1995;9:358–64. 7795640 10.1038/ng0495-358

[5] Thomas R, Sanna-Cherchi S, Warady BA, Furth SL, Kaskel FJ, Gharavi AG. HNF1B and PAX2 mutations are a common cause of renal hypodysplasia in the CKiD cohort. Pediatr Nephrol. 2011;26:897–903. 21380624 10.1007/s00467-011-1826-9 PMC3257470

[6] Hwang D-Y, Dworschak GC, Kohl S, Saisawat P, Vivante A, Hilger AC, et al. Mutations in 12 known dominant disease-causing genes clarify many congenital anomalies of the kidney and urinary tract. Kidney Int. 2014;85:1429–33. 24429398 10.1038/ki.2013.508 PMC4040148

[7] Ishiwa S, Sato M, Morisada N, Nishi K, Kanamori T, Okutsu M, et al. Association between the clinical presentation of congenital anomalies of the kidney and urinary tract (CAKUT) and gene mutations: an analysis of 66 patients at a single institution. Pediatr Nephrol. 2019;34:1457–64. 30937553 10.1007/s00467-019-04230-w

[8] Nicolaou N, Pulit SL, Nijman IJ, Monroe GR, Feitz WF, Schreuder MF, et al. Prioritization and burden analysis of rare variants in 208 candidate genes suggest they do not play a major role in CAKUT. Kidney Int. 2016;89:476–86. 26489027 10.1038/ki.2015.319

[9] Barua M, Stellacci E, Stella L, Weins A, Genovese G, Muto V, et al. Mutations in PAX2 associate with adult-onset FSGS. J Am Soc Nephrol. 2014;25:1942–53. 24676634 10.1681/ASN.2013070686 PMC4147972

[10] Okumura T, Furuichi K, Higashide T, Sakurai M, Hashimoto S-i, Shinozaki Y, et al. Association of PAX2 and other gene mutations with the clinical manifestations of renal coloboma syndrome. PLoS ONE. 2015;10:e0142843. 26571382 10.1371/journal.pone.0142843 PMC4646464

[11] Vivante A, Chacham OS, Shril S, Schreiber R, Mane SM, Pode-Shakked B, et al. Dominant PAX2 mutations may cause steroid-resistant nephrotic syndrome and FSGS in children. Pediatr Nephrol. 2019;34:1607–13. 31001663 10.1007/s00467-019-04256-0 PMC6660980

[12] Ottlewski I, Münch J, Wagner T, Schönauer R, Bachmann A, Weimann A, et al. Value of renal gene panel diagnostics in adults waiting for kidney transplantation due to undetermined end-stage renal disease. Kidney Int. 2019;96:222–30. 31027891 10.1016/j.kint.2019.01.038

[13] Bullich G, Domingo-Gallego A, Vargas I, Ruiz P, Lorente-Grandoso L, Furlano M, et al. A kidney-disease gene panel allows a comprehensive genetic diagnosis of cystic and glomerular inherited kidney diseases. Kidney Int. 2018;94:363–71. 29801666 10.1016/j.kint.2018.02.027

[14] Hays T, Groopman EE, Gharavi AG. Genetic testing for kidney disease of unknown etiology. Kidney Int. 2020;98:590–600. 32739203 10.1016/j.kint.2020.03.031 PMC7784921

[15] Porteous S, Torban E, Cho N-P, Cunliffe H, Chua L, McNoe L, et al. Primary renal hypoplasia in humans and mice with PAX2 mutations: evidence of increased apoptosis in fetal kidneys of Pax2 1Neu+/–mutant mice. Hum Mol Genet. 2000;9:1–11. 10587573 10.1093/hmg/9.1.1

[16] Dziarmaga A, Clark P, Stayner C, Julien JP, Torban E, Goodyer P, et al. Ureteric bud apoptosis and renal hypoplasia in transgenic PAX2-Bax fetal mice mimics the renal-coloboma syndrome. J Am Soc Nephrol. 2003;14:2767–74. 14569086 10.1097/01.asn.0000094082.11026.ee

[17] Dziarmaga A, Eccles M, Goodyer P. Suppression of ureteric bud apoptosis rescues nephron endowment and adult renal function in Pax2 mutant mice. J Am Soc Nephrol. 2006;17:1568–75. 16672320 10.1681/ASN.2005101074

[18] Yang Y, Jeanpierre C, Dressler GR, Lacoste M, Niaudet P, Gubler M-C. WT1 and PAX-2 podocyte expression in Denys-Drash syndrome and isolated diffuse mesangial sclerosis. Am J Pathol. 1999;154:181–92. 9916932 10.1016/S0002-9440(10)65264-9 PMC1853439

[19] Wagner K-D, Wagner N, Guo J-K, Elger M, Dallman MJ, Bugeon L, et al. An inducible mouse model for PAX2-dependent glomerular disease: insights into a complex pathogenesis. Curr Biol. 2006;16:793–800. 16631587 10.1016/j.cub.2006.02.072

[20] Deng H, Zhang Y, Xiao H, Yao Y, Liu X, Su B, et al. Diverse phenotypes in children with PAX2‐related disorder. Mol Genet Genom Med. 2019;7:e701. 10.1002/mgg3.701 PMC6565600 31060108

[21] Rossanti R, Morisada N, Nozu K, Kamei K, Horinouchi T, Yamamura T, et al. Clinical and genetic variability of PAX2-related disorder in the Japanese population. J Hum Genet. 2020;65:541–9. 32203253 10.1038/s10038-020-0741-y

[22] Ahn YH, Lee C, Kim NK, Park E, Kang HG, Ha I-S, et al. Targeted exome sequencing provided comprehensive genetic diagnosis of congenital anomalies of the kidney and urinary tract. J Clin Med. 2020;9:751. 32164334 10.3390/jcm9030751 PMC7141392

[23] Park E, Lee C, Kim NK, Ahn YH, Park YS, Lee JH, et al. Genetic study in Korean pediatric patients with steroid-resistant nephrotic syndrome or focal segmental glomerulosclerosis. J Clin Med. 2020;9:2013. 32604935 10.3390/jcm9062013 PMC7355646

[24] Park HC, Ryu H, Kim Y-C, Ahn C, Lee K-B, Kim YH, et al. Genetic identification of inherited cystic kidney diseases for implementing precision medicine: a study protocol for a 3-year prospective multicenter cohort study. BMC Nephrol. 2021;22:1–8. 33407230 10.1186/s12882-020-02207-8 PMC7786983

[25] Cheong HI, Cho HY, Kim JH, Yu YS, Ha IS, Choi Y. A clinico-genetic study of renal coloboma syndrome in children. Pediatr Nephrol. 2007;22:1283–9. 17541647 10.1007/s00467-007-0525-z

[26] Jung J, Lee JH, Park YS, Seo GH, Keum C, Kang HG, et al. Ultra-rare renal diseases diagnosed with whole-exome sequencing: Utility in diagnosis and management. BMC Med Genom. 2021;14:177. 10.1186/s12920-021-01026-6 PMC8254264 34217267

[27] Schwartz GJ, Work DF. Measurement and estimation of GFR in children and adolescents. Clin J Am Soc Nephrol. 2009;4:1832–43. 19820136 10.2215/CJN.01640309

[28] Inker LA, Eneanya ND, Coresh J, Tighiouart H, Wang D, Sang Y, et al. New creatinine-and cystatin C–based equations to estimate GFR without race. N. Engl J Med. 2021;385:1737–49. 34554658 10.1056/NEJMoa2102953 PMC8822996

[29] Parsa CF, Silva ED, Sundin OH, Goldberg MF, De Jong MR, Sunness JS, et al. Redefining papillorenal syndrome: an underdiagnosed cause of ocular and renal morbidity. Ophthalmology. 2001;108:738–49. 11297491 10.1016/s0161-6420(00)00661-8

[30] Yang X, Li Y, Fang Y, Shi H, Xiang T, Liu J, et al. Phenotypic spectrum and genetics of PAX2-related disorder in the Chinese cohort. BMC Med Genom. 2021;14:1–14. 10.1186/s12920-021-01102-x PMC8543950 34696790

[31] Savell J, Cook JR. Optic nerve colobomas of autosomal-dominant heredity. Arch Ophthalmol. 1976;94:395–400. 945057 10.1001/archopht.1976.03910030183002

[32] Parsa CF, Parsa A. Diagnosing papillorenal syndrome: see the optic papilla. Pediatr Nephrol. 2008;23:1893–4. 18512081 10.1007/s00467-008-0870-6

[33] Negrisolo S, Benetti E. PAX2 and CAKUT phenotypes: report on two New variants and a review of mutations from the Leiden Open Variation Database. Int J Mol Sci. 2023;24:4165. 36835576 10.3390/ijms24044165 PMC9962628

[34] Xiong H-Y, Shi Y-Q, Zhong C, Yang Q, Zhang G, Yang H, et al. Detection of de novo PAX2 variants and phenotypes in Chinese population: a single-center study. Front Genet. 2022;13:799562. 35444690 10.3389/fgene.2022.799562 PMC9014304

[35] Taranta A, Palma A, De Luca V, Romanzo A, Massella L, Emma F. Renal-coloboma syndrome: a single nucleotide deletion in the PAX2 gene at Exon 8 is associated with a highly variable phenotype. Clin Nephrol. 2007;67:1–4. 17269592 10.5414/cnp67001

[36] Chang Y-M, Chen C-C, Lee N-C, Sung J-M, Chou Y-Y, Chiou Y-Y. PAX2 mutation-related renal hypodysplasia: review of the literature and three case reports. Front Pediatr. 2022;9:765929. 35087773 10.3389/fped.2021.765929 PMC8787321

[37] Bower M, Salomon R, Allanson J, Antignac C, Benedicenti F, Benetti E, et al. Update of PAX2 mutations in renal coloboma syndrome and establishment of a locus‐specific database. Hum Mutat. 2012;33:457–66. 22213154 10.1002/humu.22020 PMC12123156

[38] Alur RP, Vijayasarathy C, Brown JD, Mehtani M, Onojafe IF, Sergeev YV, et al. Papillorenal syndrome-causing missense mutations in PAX2/Pax2 result in hypomorphic alleles in mouse and human. PLoS Genet. 2010;6:e1000870. 20221250 10.1371/journal.pgen.1000870 PMC2832668

[39] Cross SH, McKie L, West K, Coghill EL, Favor J, Bhattacharya S, et al. The Opdc missense mutation of Pax2 has a milder than loss-of-function phenotype. Hum Mol Genet. 2011;20:223–34. 20943750 10.1093/hmg/ddq457 PMC3005898

[40] Yamamura Y, Furuichi K, Murakawa Y, Hirabayashi S, Yoshihara M, Sako K, et al. Identification of candidate PAX2-regulated genes implicated in human kidney development. Sci Rep. 2021;11:9123. 33907292 10.1038/s41598-021-88743-1 PMC8079710

[41] Izzedine H, Bodaghi B, Launay-Vacher V, Deray G. Eye and kidney: from clinical findings to genetic explanations. J Am Soc Nephrol. 2003;14:516–29. 12538754 10.1097/01.asn.0000051705.97966.ad

[42] Preston R, Stuart HM, Lennon R. Genetic testing in steroid-resistant nephrotic syndrome: why, who, when and how? Pediatr Nephrol. 2019;34:195–210. 29181713 10.1007/s00467-017-3838-6 PMC6311200

[43] Bekheirnia MR, Bekheirnia N, Bainbridge MN, Gu S, Coban Akdemir ZH, Gambin T, et al. Whole-exome sequencing in the molecular diagnosis of individuals with congenital anomalies of the kidney and urinary tract and identification of a new causative gene. Genet Med. 2017;19:412–20. 27657687 10.1038/gim.2016.131 PMC5362362

[44] Iwafuchi Y, Morioka T, Morita T, Yanagihara T, Oyama Y, Morisada N, et al. Diverse renal phenotypes observed in a single family with a genetic mutation in paired box protein 2. Case Rep. Nephrol Dialysis. 2016;6:61–9. 10.1159/000445679 PMC4870939 27226968

[45] Schulze TG, McMahon FJ. Defining the phenotype in human genetic studies: forward genetics and reverse phenotyping. Hum Heredity. 2005;58:131–8. 10.1159/000083539 15812169

[46] Landini S, Mazzinghi B, Becherucci F, Allinovi M, Provenzano A, Palazzo V, et al. Reverse phenotyping after whole-exome sequencing in steroid-resistant nephrotic syndrome. Clin J Am Soc Nephrol. 2020;15:89–100. 31831576 10.2215/CJN.06060519 PMC6946071

[47] Wühl E, van Stralen KJ, Verrina E, Bjerre A, Wanner C, Heaf JG, et al. Timing and outcome of renal replacement therapy in patients with congenital malformations of the kidney and urinary tract. Clin J Am Soc Nephrol. 2013;8:67–74. 23085722 10.2215/CJN.03310412 PMC3531653

[48] Warady BA, Abraham AG, Schwartz GJ, Wong CS, Muñoz A, Betoko A, et al. Predictors of rapid progression of glomerular and nonglomerular kidney disease in children and adolescents: the chronic kidney disease in children (CKiD) cohort. Am J Kidney Dis. 2015;65:878–88. 25799137 10.1053/j.ajkd.2015.01.008 PMC4578873

