# Supplementary material for: Genotype of PAX2-related disorders correlates with kidney and ocular manifestations
Source: Eur J Hum Genet. 2025 Feb 24;33(4):441–50. doi: 10.1038/s41431-025-01822-z (PMC11986020; doi:10.1038/s41431-025-01822-z)
Supplement: Supplementary file 1 — SUPPLEMENTAL MATERIAL [file 41431_2025_1822_MOESM1_ESM.pdf]

SUPPLEMENTAL MATERIAL

Supplementary Table S1. Genetic diagnosis of patients with *PAX2* pathogenic variants

Supplementary Table S2. Targeted and whole exome sequencing methods

Supplementary Table S3. Genotypic and phenotypic data of *PAX2* pathogenic variants from published data to date (including this study) (n=328)

Supplementary Fig. S1. Synopsis of the clinical course in patients with *PAX2* pathogenic variants

Supplementary Table S1. Genetic diagnosis of patients with *PAX2* pathogenic variants

Case	cDNA	Protein	Segregation	Frequency in gnomAD	Mutation taster	ACMG classification[a]	ACMG criteria	Genetic test	Age at genetic diagnosis (years)	Previ-ously publis-hed
Renal coloboma syndrome										
1	c.76dupG	p.Val26Glyfs*28	Maternal germline	0	DC	Pathogenic	PVS1, PM2, PM6, PP5	Sanger	20.0	1
2	c.76dupG	p.Val26Glyfs*28	Maternal germline	0	DC	Pathogenic	PVS1, PM2, PM6, PP5	Sanger	16.5	1
3	c.76dupG	p.Val26Glyfs*28	De novo	0	DC	Pathogenic	PVS1, PM2, PM6, PP5	Sanger	12.8	1
4	c.76dupG	p.Val26Glyfs*28	De novo	0	DC	Pathogenic	PVS1, PM2, PM6, PP5	Sanger	13.9	1
5	c.76dupG	p.Val26Glyfs*28	De novo	0	DC	Pathogenic	PVS1, PM2, PM6, PP5	Sanger	1.9	1
6	c.310C>T	p.Arg104*	De novo	0	DC	Pathogenic	PVS1, PM2, PM6, PP5	Sanger	9.9	1
7	c.754C>T	p.Arg252*	De novo	0	DC	Pathogenic	PVS1, PM2, PM6, PP5	Sanger	15.1	-
8	c.76dupG	p.Val26Glyfs*28	De novo	0	DC	Pathogenic	PVS1, PM2, PM6, PP5	TES	15.1	2
9	c.344G>C	p.Arg115Pro	NA	0	DC	Likely pathogenic	PM1, PM2, PP3, PP5	TES	19.0	2
10	c.535_546delinsT	p.Asn179Trpfs*17	NA	0	DC	Likely pathogenic	PVS1, PM2	TES	4.2	-
11	c.76dupG	p.Val26Glyfs*28	NA	0	DC	Pathogenic	PVS1, PM2, PP5	Sanger	0.3	-
12	c.76dupG	p.Val26Glyfs*28	NA	0	DC	Pathogenic	PVS1, PM2, PP5	WES	5.3	-
13	c.343C>T	p.Arg115*	De novo	0	DC	Pathogenic	PVS1, PM2, PM6, PP5	TES	10.0	-
14	c.860delA	p.Gln287Argfs*10	NA	0	DC	Pathogenic	PVS1, PM2, PP5	WES	10.5	-
15	c.754C>T	p.Arg252*	Paternal	0	DC	Pathogenic	PVS1, PM2, PP1, PP5	TES	11.9	-
16	c.76dupG	p.Val26Glyfs*28	NA	0	DC	Pathogenic	PVS1, PM2, PP5	WES	5.4	-
17	c.76delG	p.Val26Cysfs*3	NA	0	DC	Pathogenic	PVS1, PM2, PP5	WES	16.0	-
18	c.76dupG	p.Val26Glyfs*28	NA	0	DC	Pathogenic	PVS1, PM2, PP5	WES	42.4	-
19	c.361_373dup	p.Asp125Glyfs*5	NA	0	DC	Likely pathogenic	PVS1, PM2	TES	32.8	-
Focal segmental glomerulosclerosis										
20	c.76dupG	p.Val26Glyfs*28	NA	0	DC	Pathogenic	PVS1, PM2, PP5	TES	26.3	3
21	c.223_226dup	p.Gly76Aspfs*27	De novo	0	DC	Pathogenic	PVS1, PM2, PM6	TES	24.6	3
22	c.74G>A	p.Gly25Glu	NA	0	DC	Likely pathogenic	PM1, PM2, PM5, PP3	TES	23.1	3
23	c.419G>A	p.Arg140Gln	NA	0	DC	Likely pathogenic	PM1, PM2, PM5, PP3	TES	10.9	3
Isolated congenital anomalies of the kidney and urinary tract										
24	c.686-1G>T	-	Maternal	0	DC	Pathogenic	PVS1, PM2, PP1	WES	10.5	4
25	c.832C>T	p.Gln278*	NA	0	DC	Likely pathogenic	PVS1, PM2	TES	8.8	-
26	c.1052G>T	p.Gly351Val	Paternal	0	DC	Likely pathogenic	PM1, PM2, PP1, PP3	TES	0.9	-

27	c.206T>C	p.Leu69Pro	NA	0	DC	Likely pathogenic	PM1, PM2, PP3, PP5	WES	30.4	-

RCS, renal coloboma syndrome; FSGS, focal segmental glomerulosclerosis; RHD, renal hypodyplasia; NA, not available; gnomAD, Genome Aggregation Database; ACMG, American College of Medicine Genetics; DC, disease-causing; TES, targeted exome sequencing; WES, whole exome sequencing; PVS, pathogenic very strong; PM, pathogenic moderate; PP, pathogenic supporting;

The reference sequence of *PAX2* gene is NM_003990.5

[a]Pathogenicity according to ACMG guidelines: pathogenic criterion; very strong (PVS1), strong (PS1-4), moderate (PM1-6), or supporting (PP1-5), and benign criterion; strong (BS1-4) or supporting (BP1-6)

Supplementary Table S2. Targeted and whole exome sequencing methods

	Covered genes	Exome capture	Sequencing	Human reference genome
**TES**				
**CAKUT**	60	Twist custom panel (Twist Bioscience, San Francisco, CA, USA)	MiSeq platform (Illumina, San Diego, CA, USA)	GRCh37/hg19
**SRNS**	57	SureSelect XT customized kit (Agilent Technologies, Santa Clara, CA, USA)	HiSeq 2500 platform (Illumina, San Diego, CA, USA)	GRCh37/hg19
**Cystic kidney disease**	89	Targeted gene panel (Twist Bioscience, San Francisco, CA, USA)	NovaSeq 6000 platform (Illumina, San Diego, CA, USA)	GRCh37/hg19
**WES**	all human genes (approximately 22,000)	SureSelect kit (Version C2; Agilent Technologies, Santa Clara, CA, USA)	NovaSeq 6000 platform (Illumina, San Diego, CA, USA)	GRCh37/hg19

TES, targeted exome sequencing; CAKUT, congenital anomalies of the kidney and urinary tract; SRNS, steroid-resistant nephrotic syndrome; WES, whole exome

Supplementary Table S3. Genotypic and phenotypic data of *PAX2* pathogenic variants from published data to date (including this study)

ID	Category	cDNA change	Protein change	Variant type	Ocular involvement	Onset or diagnosis age (year)	Kidney outcome and age at last visit (year)	Ref. PMID
1	CAKUT	c.5A>G	p.Asp2Gly	Missense	No	2	NA	Barua et al. (2014) PMID: 24676634
2	CAKUT	c.43+1G>C	-	Splicing	No	Prenatal	Normal	Bower et al. (2012) PMID: 22213154
3	RCS	c.43+1G>A	-	Splicing	Yes	8	NA	Yang et al. (2021) PMID: 34696790
4	RCS	c.51del	p.His17Glnfs*4	Frameshift	Yes	2	CKD G3b (2)	Rossanti et al. (2020) PMID: 32203253
5	RCS	c.58_64dup	p.Gln22Argfs*34	Frameshift	Yes	14	CKD G2 (14)	Okumura et al. (2015) PMID: 26571382
6	RCS	c.59del	p.Val20Glyfs*9	Frameshift	Yes	0.3	KF (5)	Chung et al. (2001) PMID: 11730657
7	RCS	c.58_64dup	p.Gln22Argfs*34	Frameshift	Yes	NA	CKD G2 (14)	Okumura et al. (2015) PMID: 26571382
8	RCS	c.68del	p.Leu23Profs*6	Frameshift	Yes	NA	NA	Cunliffe et al. (1998) PMID: 9783702
9	CAKUT	c.69dup	p.Leu24Alafs*104	Frameshift	NA	NA	CKD G4 (7.7)	Thomas et al. (2011) PMID: 21380624
10	CAKUT	c.69del	p.Val26Cys*3	Frameshift	No	Childhood	KF (NA)	Negrisolo et al. (2011) PMID: 21108633
11	RCS	c.69delC	p.Val26Cysfs*2	Frameshift	Yes	NA	NA	Mansilla et al. (2021) PMID: 31738409
12	RCS	c.69delC	p.Val26Cysfs*2	Frameshift	Yes	NA	NA	Negrisolo et al. (2023) PMID: 36835576
13	CAKUT	c.69delC	p.Leu23Leufs*6	Frameshift	NA	17	KF (17)	Yang et al. (2021) PMID: 34696790
14	RCS	c.70dupG	p.Leu23fs	Frameshift	Yes	19	KF (9)	Chang et al. (2022) PMID: 35087773
15	RCS	c.70delG	p.Gly24Alafs*8	Frameshift	Yes	NA	NA	Bekheirnia et al. (2017) PMID: 27657687
16	CAKUT	c.70G>C	p.Gly24Arg	Missense	No	3	CKD G2 (3)	Rossanti et al. (2020) PMID: 32203253
17	CKD	c.70G>C	p.Gly24Arg	Missense	NA	32	CKD (NA)	Rossanti et al. (2020) PMID: 32203253
18	CAKUT	c.70G>T	p.Gly24Trp	Missense	NA	0.2	CKD G3b (3.4)	Xiong et al. (2022) PMID: 35444690
19	RCS/FSGS	c.70_72delinsA	p.Gly24Argfs*29	Frameshift	Yes	6	KF (8)	Saida et al. (2020) PMID: 31538321
20	FSGS	c.70_71insG	p.Val26Glyfx*28	Frameshift	No	NA	NA	Mansilla et al. (2021) PMID: 31738409
21	CAKUT	c.71G>A	p.Gly24Glu	Missense	NA	NA	CKD G3b (15.4)	Thomas et al. (2011) PMID: 21380624
22	FSGS	c.74G>A	p.Gly25Glu	Missense	No	7.3	KF (14.5)	This study
23	RCS	c.74G>T	p.Gly25Val	Missense	Yes	NA	KF (23)	Bower et al. (2012) PMID: 22213154

24	RCS	c.74G>T	p.Gly25Val	Missense	Yes	NA	CKD (NA)	Bower et al. (2012) PMID: 22213154
25	RCS	c.75_76dup	p.Val26Glyfs*4	Frameshift	Yes	NA	NA	Amiel et al. (2000) PMID: 11093271
26	RCS	c.76dupG	p.Val26Glyfs*28	Frameshift	Yes	12	KF (13)	Cheong et al. (2007) PMID: 17541647
27	RCS	c.76dupG	p.Val26Glyfs*28	Frameshift	Yes	3	KF (17)	Cheong et al. (2007) PMID: 17541647
28	RCS/FSGS	c.76dupG	p.Val26Glyfs*28	Frameshift	Yes	2	CKD G3 (11)	Cheong et al. (2007) PMID: 17541647
29	RCS	c.76dupG	p.Val26Glyfs*28	Frameshift	Yes	7	KF (9)	Cheong et al. (2007) PMID: 17541647
30	RCS	c.76dupG	p.Val26Glyfs*28	Frameshift	Yes	1	CKD G3 (2.5)	Cheong et al. (2007) PMID: 17541647
31	RCS	c.76dupG	p.Val26Glyfs*28	Frameshift	Yes	Neonatal	NA	Fujioka et al. (2011) PMID: 21696512
32	RCS	c.76dupG	p.Val26Glyfs*28	Frameshift	Yes	3	CKD G3b (6)	Yoshimura et al. (2005) PMID: 15808183
33	RCS	c.76dupG	p.Val26Glyfs*28	Frameshift	Yes	15	NA	Weber et al. (2006) PMID: 16971658
34	RCS	c.76dupG	p.Val26Glyfs*28	Frameshift	Yes	17	NA	Weber et al. (2006) PMID: 16971658
35	CAKUT	c.76dupG	p.Val26Glyfs*28	Frameshift	No	NA	NA	Weber et al. (2006) PMID: 16971658
36	RCS	c.76dupG	p.Val26Glyfs*28	Frameshift	Yes	14	NA	Weber et al. (2006) PMID: 16971658
37	RCS	c.76dupG	p.Val26Glyfs*28	Frameshift	Yes	10	NA	Weber et al. (2006) PMID: 16971658
38	RCS	c.76dupG	p.Val26Glyfs*28	Frameshift	Yes	4	KF (5.5)	Salomon et al. (2001) PMID: 11168927
39	RCS	c.76dupG	p.Val26Glyfs*28	Frameshift	Yes	Neonatal	KF (7)	Salomon et al. (2001) PMID: 11168927
40	RCS	c.76dupG	p.Val26Glyfs*28	Frameshift	Yes	13	CKD G3a (13)	Rossanti et al. (2020) PMID: 32203253
41	RCS	c.76dupG	p.Val26Glyfs*28	Frameshift	Yes	NA	NA	Rossanti et al. (2020) PMID: 32203253
42	CKD	c.76dupG	p.Val26Glyfs*28	Frameshift	No	25	KF (7)	Rossanti et al. (2020) PMID: 32203253
43	FSGS	c.76dupG	p.Val26Glyfs*28	Frameshift	No	51	NA	Rossanti et al. (2020) PMID: 32203253
44	CKD	c.76dupG	p.Val26Glyfs*28	Frameshift	No	26	KF (8)	Rossanti et al. (2020) PMID: 32203253
45	CAKUT	c.76dupG	p.Val26Glyfs*28	Frameshift	No	10	KF (11)	Rossanti et al. (2020) PMID: 32203253
46	RCS	c.76dupG	p.Val26Glyfs*28	Frameshift	Yes	2	CKD G3a (2)	Rossanti et al. (2020) PMID: 32203253
47	RCS	c.76dupG	p.Val26Glyfs*28	Frameshift	Yes	NA	NA	Rossanti et al. (2020) PMID: 32203253
48	RCS	c.76dupG	p.Val26Glyfs*28	Frameshift	Yes	26	KF (7)	Rossanti et al. (2020) PMID: 32203253
49	CKD	c.76dupG	p.Val26Glyfs*28	Frameshift	No	0.17	CKD	Rossanti et al. (2020) PMID: 32203253
50	RCS	c.76dupG	p.Val26Glyfs*28	Frameshift	Yes	6	CKD G3a (13)	Ohtsubo et al. (2012) PMID: 22350371

51	RCS	c.76dupG	p.Val26Glyfs*28	Frameshift	Yes	0.33	CKD	Ohtsubo et al. (2012) PMID: 22350371
52	RCS	c.76dupG	p.Val26Glyfs*28	Frameshift	Yes	3	CKD	Rossanti et al. (2020) PMID: 32203253
53	CAKUT	c.76dupG	p.Val26Glyfs*28	Frameshift	No	9	CKD G3b (9)	Rossanti et al. (2020) PMID: 32203253
54	RCS	c.76dupG	p.Val26Glyfs*28	Frameshift	Yes	20	CKD G3 (52)	Iwafuchi et al. (2016) PMID: 27226968
55	RCS	c.76dupG	p.Val26Glyfs*28	Frameshift	Yes	2	KF (5)	Iwafuchi et al. (2016) PMID: 27226968
56	RCS	c.76dupG	p.Val26Glyfs*28	Frameshift	Yes	Neonatal	KF (7)	Iwafuchi et al. (2016) PMID: 27226968
57	RCS	c.76dupG	p.Val26Glyfs*28	Frameshift	Yes	NA	CKD G2 (NA)	Sanyanusin et al. (1995) PMID: 8589702
58	RCS	c.76dupG	p.Val26Glyfs*28	Frameshift	Yes	NA	KF (NA)	Sanyanusin et al. (1995) PMID: 8589702
59	RCS	c.76dupG	p.Val26Glyfs*28	Frameshift	Yes	48	KF (24)	Schimmenti et al. (1997) PMID: 9106533
60	RCS	c.76dupG	p.Val26Glyfs*28	Frameshift	Yes	25	KF (14)	Schimmenti et al. (1997) PMID: 9106533
61	RCS	c.76dupG	p.Val26Glyfs*28	Frameshift	Yes	3	KF (18)	Schimmenti et al. (1997) PMID: 9106533
62	RCS	c.76dupG	p.Val26Glyfs*28	Frameshift	Yes	Neonatal	CKD G2-3a (7)	Ford et al. (2001) PMID: 11241473
63	RCS	c.76dupG	p.Val26Glyfs*28	Frameshift	Yes	4	KF (23)	Ford et al. (2001) PMID: 11241473
64	RCS	c.76dupG	p.Val26Glyfs*28	Frameshift	Yes	NA	CKD G3a (37)	Ford et al. (2001) PMID: 11241473
65	CKD	c.76dupG	p.Val26Glyfs*28	Frameshift	No	35	KF (60)	Ford et al. (2001) PMID: 11241473
66	RCS	c.76dupG	p.Val26Glyfs*28	Frameshift	Yes	NA	NA	Amiel et al. (2000) PMID: 11093271
67	RCS	c.76dupG	p.Val26Glyfs*28	Frameshift	Yes	NA	NA	Amiel et al. (2000) PMID: 11093271
68	RCS	c.76dupG	p.Val26Glyfs*28	Frameshift	Yes	NA	NA	Amiel et al. (2000) PMID: 11093271
69	RCS	c.76dupG	p.Val26Glyfs*28	Frameshift	Yes	NA	NA	Amiel et al. (2000) PMID: 11093271
70	RCS	c.76dupG	p.Val26Glyfs*28	Frameshift	Yes	NA	NA	Amiel et al. (2000) PMID: 11093271
71	RCS	c.76dupG	p.Val26Glyfs*28	Frameshift	Yes	Neonatal	KF (3)	Schimmenti et al. (1999) PMID: 10533062
72	RCS	c.76dupG	p.Val26Glyfs*28	Frameshift	Yes	1	KF (4)	Vivante et al. (2019) PMID: 31001663
73	FSGS	c.76dupG	p.Val26Glyfs*28	Frameshift	No	14	CKD G3b (14)	Rossanti et al. (2020) PMID: 32203253
74	CAKUT	c.76dupG	p.Val26Glyfs*28	Frameshift	No	13.2	KF (13.2)	Xiong et al. (2022) PMID: 35444690
75	RCS	c.76dupG	p.Val26Glyfs*28	Frameshift	Yes	9.7	CKD G3a (10.4)	Xiong et al. (2022) PMID: 35444690
76	CAKUT	c.76dupG	p.Val26Glyfs*28	Frameshift	NA	Prenatal	NA	Xiong et al. (2022) PMID: 35444690
77	RCS	c.76dupG	p.Val26Glyfs*28	Frameshift	Yes	0.02	KF (0)	Chang et al. (2022) PMID: 35087773

78	RCS	c.76dupG	p.Val26Glyfs*28	Frameshift	Yes	34	CKD G3 (34)	Chang et al. (2022) PMID: 35087773
79	NA	c.76dupG	p.Val26Glyfs*28	Frameshift	NA	NA	KF (NA)	Bower et al. (2012) PMID: 22213154
80	RCS	c.76dupG	p.Val26Glyfs*28	Frameshift	Yes	7.2	KF (12.2)	This study
81	RCS	c.76dupG	p.Val26Glyfs*28	Frameshift	Yes	3.8	KF (17.5)	This study
82	RCS	c.76dupG	p.Val26Glyfs*28	Frameshift	Yes	0.2	KF (14.8)	This study
83	RCS	c.76dupG	p.Val26Glyfs*28	Frameshift	Yes	6.6	KF (8.6)	This study
84	RCS	c.76dupG	p.Val26Glyfs*28	Frameshift	Yes	0.5	KF (7.1)	This study
85	RCS	c.76dupG	p.Val26Glyfs*28	Frameshift	Yes	0.1	KF (3.2)	This study
86	RCS	c.76dupG	p.Val26Glyfs*28	Frameshift	Yes	0.1	CKD G4 (13.1)	This study
87	RCS	c.76dupG	p.Val26Glyfs*28	Frameshift	Yes	0.1	CKD G4 (6.4)	This study
88	RCS	c.76dupG	p.Val26Glyfs*28	Frameshift	Yes	0.1	CKD G3a (5.4)	This study
89	RCS	c.76dupG	p.Val26Glyfs*28	Frameshift	Yes	10.2	KF (12.9)	This study
90	FSGS/ocular phenotype	c.76dupG	p.Val26Glyfs*28	Frameshift	Yes	5.2	KF (15.5)	This study
91	RCS	c.76dupG	p.Val26Glyfs*28	Frameshift	Yes	1	KF (10)	Yang et al. (2021) PMID: 34696790
92	RCS	c.76dupG	p.Val26Glyfs*28	Frameshift	Yes	5	KF (5)	Yang et al. (2021) PMID: 34696790
93	CAKUT	c.76dupG	p.Val26Glyfs*28	Frameshift	NA	1	KF (6)	Yang et al. (2021) PMID: 34696790
94	Nephrosis	c.76dupG	p.Val26Glyfs*28	Frameshift	No	6.8	CKD G4 (NA)	Yang et al. (2021) PMID: 34696790
95	Nephrosis	c.76dupG	p.Val26Glyfs*28	Frameshift	No	8.2	CKD G4 (NA)	Yang et al. (2021) PMID: 34696790
96	RCS	c.76dupG	p.Val26Glyfs*28	Frameshift	Yes	13	CKD G4 (NA)	Yang et al. (2021) PMID: 34696790
97	Nephrosis	c.76dupG	p.Val26Glyfs*28	Frameshift	NA	24	CKD G3 (NA)	Yang et al. (2021) PMID: 34696790
98	Nephrosis	c.76dupG	p.Val26Glyfs*28	Frameshift	NA	22	CKD G2 (NA)	Yang et al. (2021) PMID: 34696790
99	NA	c.76del	p.Val26Cysfs*3	Frameshift	NA	1	KF (3)	Rossanti et al. (2020) PMID: 32203253
100	RCS	c.76del	p.Val26Cysfs*3	Frameshift	Yes	9	CKD G3 (9)	Rossanti et al. (2020) PMID: 32203253
101	RCS	c.76del	p.Val26Cysfs*3	Frameshift	Yes	5	NA	Schimmenti et al. (1999) PMID: 10533062
102	RCS	c.76del	p.Val26Cysfs*3	Frameshift	Yes	4	NA	Schimmenti et al. (1999) PMID: 10533062
103	RCS	c.76delG	p.Val26Cysfs*3	Frameshift	Yes	0.1	KF (0.2)	This study
104	CAKUT	c.81_103delinsC	p.Val28Thrfs*3	Frameshift	NA	1d	NA	Yang et al. (2021) PMID: 34696790

105	CAKUT	c.88G>T	p.Gly30Cys	Missense	No	9.8	KF (9.8)	Deng et al. (2019) PMID: 31060108
106	RCS	c.89G>T	p.Gly30Val	Missense	Yes	1	CKD	Rossanti et al. (2020) PMID: 32203253
107	CAKUT	c.89del	p.Gly30Alafs*8	Frameshift	No	1	CKD G3 (1)	Rossanti et al. (2020) PMID: 32203253
108	CAKUT	c.92_97del	p.Arg31_Pro32del	In-frame	No	NA	NA	Weber et al. (2006) PMID: 16971658
109	RCS	c.98T>G	p.Leu33Arg	Missense	Yes	NA	KF (5)	Bower et al. (2012) PMID: 22213154
110	RCS	c.115_120del	p.Gln39_Arg 40del	In-frame	Yes	Neonatal	KF (20)	Salomon et al. (2001) PMID: 11168927
111	RCS	c.117_118del	p.Arg40Hisfs*13	Frameshift	Yes	NA	KF (44)	Okumura et al. (2015) PMID: 26571382
112	RCS	c.117_118del	p.Arg40Hisfs*13	Frameshift	Yes	18	CKD G2 (18)	Okumura et al. (2015) PMID: 26571382
113	RCS	c.117_118del	p.Arg40Hisfs*13	Frameshift	Yes	NA	KF (61)	Okumura et al. (2015) PMID: 26571382
114	RCS	c.117_118del	p.Arg40Hisfs*13	Frameshift	Yes	NA	CKD G3 (59)	Okumura et al. (2015) PMID: 26571382
115	RCS	c.117_118del	p.Arg40Hisfs*13	Frameshift	Yes	NA	KF (61)	Okumura et al. (2015) PMID: 26571382
116	RCS	c.131_152del	p.Leu44Profs*32	Frameshift	Yes	0.25	KF (2)	Schimmenti et al. (1997) PMID: 9106533
117	RCS	c.139_148del	p.Gln47Glyfs*33	Frameshift	Yes	21	CKD G4 (29)	Fletcher et al. (2005) PMID: 16049068
118	RCS	c.139_148del	p.Gln47Glyfs*33	Frameshift	Yes	3	KF (14)	Fletcher et al. (2005) PMID: 16049068
119	RCS	c.139_148del	p.Gln47Glyfs*33	Frameshift	Yes	1.5	KF (24)	Fletcher et al. (2005) PMID: 16049068
120	RCS	c.139_148del	p.Gln47Glyfs*33	Frameshift	Yes	Prenatal	CKD G3 (3)	Fletcher et al. (2005) PMID: 16049068
121	RCS	c.139_148del	p.Gln47Glyfs*33	Frameshift	Yes	Prenatal	CKD G3 (0.5)	Fletcher et al. (2005) PMID: 16049068
122	RCS	c.139_148del	p.Gln47Glyfs*33	Frameshift	Yes	Prenatal	CKD G3 (0.5)	Fletcher et al. (2005) PMID: 16049068
123	CAKUT	c.143delG	p.Gly48Valfs*34	Frameshift	NA	2	CKD G3 (NA)	Yang et al. (2021) PMID: 34696790
124	RCS	c.143del	p.Gly48Vlafs*35	Frameshift	Yes	5	CKD G2 (5)	Rossanti et al. (2020) PMID: 32203253
125	Nephrosis	c.148C>T	p.Arg50Trp	Missense	No	23	KF (25)	Yang et al. (2021) PMID: 34696790
126	Nephrosis	c.148C>T	p.Arg50Trp	Missense	No	34	KF (37)	Yang et al. (2021) PMID: 34696790
127	Nephrosis	c.148C>T	p.Arg50Trp	Missense	No	35	KF (36)	Yang et al. (2021) PMID: 34696790
128	CAKUT	c.153_155delCTG insTT	p.Cys52Leufs*31	Frameshift	No	NA	NA	Negrisolo et al. (2023) PMID: 36835576
129	RCS	c.153_155delCTG insTT	p.Cys52Leufs*31	Frameshift	Yes	NA	NA	Negrisolo et al. (2023) PMID: 36835576
130	CAKUT	c.155G>A	p.Cys52Tyr	Missense	No	Prenatal	KF (16)	Iatropoulos et al. (2012) PMID: 22660956

131	RCS	c.155G>A	p.Cys52Tyr	Missense	Yes	Neonatal	Normal	Iatropoulos et al. (2012) PMID: 22660956
132	FSGS	c.167G>A	p.Arg56Gln	Missense	No	36	NA	Barua et al. (2014) PMID: 24676634
133	RCS	c.182G>A	p.Ser61Asn	Missense	Yes	NA	CKD	Bower et al. (2012) PMID: 22213154
134	RCS	c.182G>A	p.Ser61Asn	Missense	Yes	NA	KF (50)	Bower et al. (2012) PMID: 22213154
135	RCS	c.182G>T	p.Ser61Ile	Missense	Yes	NA	KF (9)	Bower et al. (2012) PMID: 22213154
136	RCS	c.187G>A	p.Gly63Ser	Missense	Yes	NA	CKD G4 (37)	Okumura et al. (2015) PMID: 26571382
137	RCS	c.184_198del	p.His62_Ser66del	In-frame	Yes	Neonatal	KF (0.1)	Bower et al. (2012) PMID: 22213154
138	NA	c.194T>C	p.Val65Ala	Missense	NA	NA	KF (27)	Ciccone et al. (2024) abstract
139	RCS	c.206T>C	p.Leu69Pro	Missense	Yes	NA	NA	Bower et al. (2012) PMID: 22213154
140	RCS	c.206T>C	p.Leu69Pro	Missense	Yes	NA	NA	Bower et al. (2012) PMID: 22213154
141	RCS	c.206T>C	p.Leu69Pro	Missense	Yes	NA	KF (NA)	Bower et al. (2012) PMID: 22213154
142	CAKUT	c.206T>C	p.Leu69Pro	Missense	No	9.1	KF (9.7)	This study
143	CAKUT	c.211A>G	p.Arg71Gly	Missense	NA	NA	NA	Hwang et al. (2014) PMID: 24429398
144	RCS	c.212G>C	p.Arg71Thr	Missense	Yes	15	CKD G3 (16)	Higashide et al. (2005) PMID: 15652857
145	RCS	c.212G>C	p.Arg71Thr	Missense	Yes	Childhood	KF (39)	Higashide et al. (2005) PMID: 15652857
146	RCS	c.212G>C	p.Arg71Thr	Missense	Yes	NA	KF (34)	Okumura et al. (2015) PMID: 26571382
147	RCS	c.212G>C	p.Arg71Thr	Missense	Yes	NA	KF (61)	Okumura et al. (2015) PMID: 26571382
148	RCS	c.212G>T	p.Arg71Met	Missense	Yes	0.17	CKD G4 (0.2)	Rossanti et al. (2020) PMID: 32203253
149	RCS	c.212G>T	p.Arg71Met	Missense	Yes	NA	CKD	Negrisolo et al. (2023) PMID: 36835576
150	RCS	c.213-2A>G	-	Splicing	Yes	Childhood	NA	Galvez-Ruiz et al. (2017) PMID: 29339962
151	RCS	c.213-2A>G	-	Splicing	Yes	NA	NA	Weber et al. (2006) PMID: 16971658
152	RCS	c.213-2A>G	-	Splicing	Yes	NA	KF (NA)	Weber et al. (2006) PMID: 16971658
153	CAKUT	c.213-2A>G	-	Splicing	No	9	KF (10)	Yang et al. (2021) PMID: 34696790
154	CAKUT/nephrosis	c.218_219insCGAGAC	p.Tyr73delinsTyrGluThr	In-frame	NA	9.7	CKD G2 (9.7)	Xiong et al. (2022) PMID: 35444690
155	RCS	c.219C>G	p.Tyr73*	Nonsense	Yes	NA	CKD G3-4 (17)	Bower et al. (2012) PMID: 22213154
156	RCS	c.219C>G	p.Tyr73*	Nonsense	Yes	NA	KF (35)	Bower et al. (2012) PMID: 22213154

157	CKD	c.219C>G	p.Tyr73*	Nonsense	NA	NA	KF (50)	Bower et al. (2012) PMID: 22213154
158	NA	c.219C>G	p.Tyr73*	Nonsense	NA	7	KF (12)	Xiong et al. (2022) PMID: 35444690
159	RCS	c.219C>G	p.Try73*	Nonsense	Yes	10	KF (10)	Yang et al. (2021) PMID: 34696790
160	RCS	c.220G>T	p.Glu74*	Nonsense	Yes	4	CKD G3 (4)	Rossanti et al. (2020) PMID: 32203253
161	RCS	c.221_226dup	p.Glu74_Thr75dup	In-frame	Yes	10	KF (10)	Liu et al. (2018) PMID: 29054766
162	RCS	c.221_226dup	p.Glu74_Thr75dup	In-frame	Yes	Childhood	CKD G2-3 (17)	Devriendt et al. (1998) PMID: 9760197
163	RCS	c.221_226dup	p.Glu74_Thr75dup	In-frame	Yes	Prenatal	CKD3 (NA)	Yang et al. (2021) PMID: 34696790
164	CAKUT	c.221_226dup	p.Glu74_Thr75dup	In-frame	NA	10	KF (12)	Yang et al. (2021) PMID: 34696790
165	CAKUT	c.223_225dup	p.Thr75dup	In-frame	No	NA	Normal	Bower et al. (2012) PMID: 22213154
166	RCS	c.223_225dup	p.Thr75dup	In-frame	Yes	NA	Normal	Bower et al. (2012) PMID: 22213154
167	RCS	c.223_225dup	p.Thr75dup	In-frame	Yes	NA	CKD	Bower et al. (2012) PMID: 22213154
168	RCS	c.223_225dup	p.Thr75dup	In-frame	Yes	NA	KF (36)	Bower et al. (2012) PMID: 22213154
169	CKD	c.223_225dup	p.Thr75dup	In-frame	NA	NA	KF (12)	Bower et al. (2012) PMID: 22213154
170	CKD	c.223_225dup	p.Thr75dup	In-frame	NA	NA	KF (19)	Bower et al. (2012) PMID: 22213154
171	CAKUT	c.223_225dup	p.Thr75dup	In-frame	NA	NA	NA	Bower et al. (2012) PMID: 22213154
172	CAKUT	c.223_225dup	p.Thr75dup	In-frame	NA	NA	NA	Bower et al. (2012) PMID: 22213154
173	CKD	c.223_225dup	p.Thr75dup	In-frame	NA	NA	KF (60)	Bower et al. (2012) PMID: 22213154
174	CKD	c.223_225dup	p.Thr75dup	In-frame	NA	NA	KF (79)	Bower et al. (2012) PMID: 22213154
175	CAKUT	c.223_225dup	p.Thr75dup	In-frame	NA	NA	CKD	Bower et al. (2012) PMID: 22213154
176	CAKUT	c.223_225dup	p.Thr75dup	In-frame	NA	NA	KF (19)	Bower et al. (2012) PMID: 22213154
177	RCS/FSGS	c.223_224dup	p.Gly76Profs*8	Frameshift	Yes	NA	KF (38)	Okumura et al. (2015) PMID: 26571382
178	FSGS/ocular phenotype	c.223_226dup	p.Gly76Aspfs*27	Frameshift	Yes	13.4	CKD G3b (27.5)	This study
179	RCS	c.226G>A	p.Gly76Ser	Missense	Yes	16	CKD G2 (16)	Devriendt et al. (1998) PMID: 9760197
180	RCS	c.226G>A	p.Gly76Ser	Missense	Yes	NA	KF (32)	Devriendt et al. (1998) PMID: 9760197
181	RCS	c.226G>A	p.Gly76Ser	Missense	Yes	NA	CKD G4 (46)	Devriendt et al. (1998) PMID: 9760197
182	RCS	c.226G>A	p.Gly76Ser	Missense	Yes	NA	KF (33)	Devriendt et al. (1998) PMID: 9760197
183	RCS	c.226G>A	p.Gly76Ser	Missense	Yes	NA	KF (22)	Devriendt et al. (1998) PMID: 9760197

184	RCS	c.226G>A	p.Gly76Ser	Missense	Yes	NA	CKD G3 (70)	Devriendt et al. (1998) PMID: 9760197
185	CKD	c.226G>A	p.Gly76Ser	Missense	NA	NA	KF (NA)	Devriendt et al. (1998) PMID: 9760197
186	CKD	c.226G>A	p.Gly76Ser	Missense	NA	NA	KF (NA)	Devriendt et al. (1998) PMID: 9760197
187	CKD	c.226G>A	p.Gly76Ser	Missense	NA	NA	KF (NA)	Devriendt et al. (1998) PMID: 9760197
188	CKD	c.226G>A	p.Gly76Ser	Missense	NA	NA	KF (NA)	Devriendt et al. (1998) PMID: 9760197
189	RCS	c.228_251dup	p.Ser77_Gly84dup	In-frame	Yes	NA	CKD G4 (62)	Adam et al. (2013) PMID: 27293569
190	RCS	c.228_251dup	p.Ser77_Gly84dup	In-frame	Yes	NA	KF (39)	Adam et al. (2013) PMID: 27293569
191	RCS	c.228_251dup	p.Ser77_Gly84dup	In-frame	Yes	NA	Normal	Adam et al. (2013) PMID: 27293569
192	RCS	c.228_251dup	p.Ser77_Gly84dup	In-frame	Yes	NA	Normal	Adam et al. (2013) PMID: 27293569
193	CAKUT	c.228_251dup	p.Ser77_Gly84dup	In-frame	NA	2	KF (17)	Adam et al. (2013) PMID: 27293569
194	RCS	c.239C>T	p.Pro80Leu	Missense	Yes	14	CKD G2 (14)	Rossanti et al. (2020) PMID: 32203253
195	FSGS	c.239C>T	p.Pro80Leu	Missense	No	7	NA	Barua et al. (2014) PMID: 24676634
196	FSGS	c.239C>T	p.Pro80Leu	Missense	No	11	NA	Barua et al. (2014) PMID: 24676634
197	CAKUT	c.239C>A	p.Pro80Gln	Missense	NA	22	KF (NA)	Yang et al. (2021) PMID: 34696790
198	Nephrosis	c.254G>T	p.Gly85Val	Missense	No	NA	CKD G3 (13)	Vivante et al. (2019) PMID: 31001663
199	Nephrosis	c.254G>T	p.Gly85Val	Missense	No	NA	CKD G2 (10)	Vivante et al. (2019) PMID: 31001663
200	Nephrosis	c.254G>T	p.Gly85Val	Missense	No	NA	KF (39)	Vivante et al. (2019) PMID: 31001663
201	CAKUT/nephrosis	c.272C>T	p.Ala91Val	Missense	NA	9.7	CKD G3 (11)	Deng et al. (2019) PMID: 31060108
202	nephrosis	c.275G>T	p.Thr92Met	Missense	NA	18	normal	Vivante et al. (2019) PMID: 31001663
203	CKD	c.275G>T	p.Thr92Met	Missense	NA	NA	KF (50)	Vivante et al. (2019) PMID: 31001663
204	CKD	c.275G>T	p.Thr92Met	Missense	NA	NA	KF (70)	Vivante et al. (2019) PMID: 31001663
205	RCS	c.289del	p.Asp97Thrfs*62	Frameshift	Yes	NA	NA	Amiel et al. (2000) PMID: 11093271
206	RCS	c.289del	p.Asp97Thrfs*62	Frameshift	Yes	NA	CKD	Amiel et al. (2000) PMID: 11093271
207	RCS	c.310C>T	p.Arg104*	Nonsense	Yes	0.5	KF (2)	Rossanti et al. (2020) PMID: 32203253
208	RCS	c.310C>T	p.Arg104*	Nonsense	Yes	0.17	CKD G4 (0.2)	Rossanti et al. (2020) PMID: 32203253
209	CAKUT	c.310C>T	p.Arg104*	Nonsense	No	NA	NA	Rossanti et al. (2020) PMID: 32203253
210	CAKUT	c.310C>T	p.Arg104*	Nonsense	No	0.17	NA	Rossanti et al. (2020) PMID: 32203253

211	RCS	c.310C>T	p.Arg104*	Nonsense	Yes	1	KF (10)	Cheong et al. (2007) PMID: 17541647
212	RCS/FSGS	c.310C>T	p.Arg104*	Nonsense	Yes	NA	KF (42)	Barua et al. (2014) PMID: 24676634
213	RCS	c.310C>T	p.Arg104*	Nonsense	Yes	0.33	KF (9.5)	This study
214	CAKUT	c.320C>T	p.Pro107Leu	Missense	NA	NA	NA	Hwang et al. (2014) PMID: 24429398
215	CAKUT	c.331G>A	p.Ala111Thr	Missense	No	NA	NA	Sellick et al. (2004) PMID: 15561999
216	CAKUT	c.331G>A	p.Ala111Thr	Missense	No	NA	NA	Sellick et al. (2004) PMID: 15561999
217	CAKUT	c.331G>A	p.Ala111Thr	Missense	No	NA	NA	Sellick et al. (2004) PMID: 15561999
218	CAKUT	c.331G>A	p.Ala111Thr	Missense	No	NA	NA	Sellick et al. (2004) PMID: 15561999
219	CAKUT	c.331G>A	p.Ala111Thr	Missense	No	NA	NA	Sellick et al. (2004) PMID: 15561999
220	CAKUT	c.331G>A	p.Ala111Thr	Missense	No	NA	NA	Sellick et al. (2004) PMID: 15561999
221	CAKUT	c.331G>A	p.Ala111Thr	Missense	No	NA	NA	Sellick et al. (2004) PMID: 15561999
222	CAKUT	c.331G>A	p.Ala111Thr	Missense	No	NA	NA	Sellick et al. (2004) PMID: 15561999
223	RCS	c.343C>T	p.Arg115*	Nonsense	Yes	NA	KF (6)	Bower et al. (2012) PMID: 22213154
224	RCS	c.343C>T	p.Arg115*	Nonsense	Yes	0.08	CKD G3 (0.1)	Rossanti et al. (2020) PMID: 32203253
225	CAKUT	c.343C>T	p.Arg115*	Nonsense	NA	NA	NA	Hwang et al. (2014) PMID: 24429398
226	RCS	c.343C>T	p.Arg115*	Nonsense	Yes	20	KF (25)	Schimmenti et al. (2003) PMID: 14566649
227	RCS	c.343C>T	p.Arg115*	Nonsense	Yes	19	KF (60)	Schimmenti et al. (2003) PMID: 14566649
228	CKD	c.343C>T	p.Arg115*	Nonsense	NA	NA	KF (NA)	Schimmenti et al. (2003) PMID: 14566649
229	CKD	c.343C>T	p.Arg115*	Nonsense	NA	NA	KF (NA)	Schimmenti et al. (2003) PMID: 14566649
230	CAKUT/FSGS	c.343C>T	p.Arg115*	Nonsense	No	6.3	CKD G3 (7.1)	Xiong et al. (2022) PMID: 35444690
231	RCS	c.343C>T	p.Arg115*	Nonsense	Yes	0.1	CKD G3a (11.4)	This study
232	RCS	c.344G>C	p.Arg115Pro	Missense	Yes	0.1	KF (10.4)	This study
233	NA	c.361G>A	p.Glu121Lys	Missense	NA	NA	KF (18)	Ciccone et al. (2024) abstract
234	NA	c.361G>A	p.Glu121Lys	Missense	NA	NA	KF (70)	Ciccone et al. (2024) abstract
235	NA	c.361G>A	p.Glu121Lys	Missense	NA	NA	KF (70)	Ciccone et al. (2024) abstract
236	RCS	c.361_373dup	p.Asp125Glyfs*5	Frameshift	Yes	4.5	KF (8.9)	This study
237	RCS	c.389C>A	p.Pro130His	Missense	Yes	NA	KF (12)	Miyazawa et al. (2009) PMID: 19954729

238	CAKUT	c.388C>T	p.Pro130Ser	Missense	No	20	KF (20)	Bower et al. (2012) PMID: 22213154
239	RCS	c.388C>T	p.Pro130Ser	Missense	Yes	2	NA	Bower et al. (2012) PMID: 22213154
240	RCS	c.389C>G	p.Pro130Arg	Missense	Yes	NA	KF (24)	Galvez-Ruiz et al. (2017) PMID: 29339962
241	FSGS	c.398C>T	p.Ser133Phe	Missense	No	8	KF (NA)	Barua et al. (2014) PMID: 24676634
242	FSGS	c.398C>T	p.Ser133Phe	Missense	No	NA	NA	Barua et al. (2014) PMID: 24676634
243	CAKUT	c.408del	p.Asn136Lysfs*23	Frameshift	NA	NA	NA	Hwang et al. (2014) PMID: 24429398
244	CAKUT	c.408del	p.Asn136Lysfs*23	Frameshift	NA	NA	NA	Hwang et al. (2014) PMID: 24429398
245	CAKUT	c.410+1G>A	-	Splicing	No	Neonatal	Normal	Deng et al. (2019) PMID: 31060108
246	RCS	c.410+5G>A	-	Splicing	Yes	NA	KF (NA)	Negrisolo et al. (2011) PMID: 21108633
247	CAKUT	c.411-1G>T	-	Splicing	NA	NA	CKD G3b (13.7)	Thomas et al. (2011) PMID: 21380624
248	CAKUT	c.415A>G	p.Ile139Val	Missense	No	NA	NA	Barua et al. (2014) PMID: 24676634
249	RCS	c.418C>T	p.Arg140Trp	Missense	Yes	14	Normal	Rachwani et al. (2019) PMID: 31692565
250	CKD	c.418C>T	p.Arg140Trp	Missense	No	1	CKD G3a (1)	Rossanti et al. (2020) PMID: 32203253
251	CAKUT	c.418C>G	p.Arg140Gly	Missense	No	Prenatal	CKD G3 (3)	Zhang et al. (2018) PMID: 30241513
252	CKD	c.418C>G	p.Arg140Gly	Missense	No	NA	KF (20)	Zhang et al. (2018) PMID: 30241513
253	RCS	c.419G>A	p.Arg140Gln	Missense	Yes	12	CKD G4 (12)	Rossanti et al. (2020) PMID: 32203253
254	FSGS	c.419G>A	p.Arg140Gln	Missense	No	7.8	CKD G2 (11.6)	This study
255	FSGS	c.419G>T	p.Arg140Leu	Missense	No	NA	NA	Mansilla et al. (2021) PMID: 31738409
256	FSGS	c.430C>T	p.Gln144*	Nonsense	No	16	CKD G3 (25.5)	Bitó et al. (2020) PMID: 33363218
257	RCS	c.432del	p.Gln144Hisfs*15	Frameshift	Yes	0.42	CKD G4 (0.4)	Rossanti et al. (2020) PMID: 32203253
258	CAKUT	c.445C>T	p.Pro149Ser	Missense	NA	14	CKD G3 (NA)	Yang et al. (2021) PMID: 34696790
259	FSGS	c.448A>G	p.Thr150Ala	Missense	No	31-32	KF (30-36)	Barua et al. (2014) PMID: 24676634
260	FSGS	c.448A>G	p.Thr150Ala	Missense	NA	NA	KF (30-36)	Barua et al. (2014) PMID: 24676634
261	FSGS	c.448A>G	p.Thr150Ala	Missense	No	NA	KF (30-36)	Barua et al. (2014) PMID: 24676634
262	CKD	c.448A>G	p.Thr150Ala	Missense	NA	NA	KF (30-36)	Barua et al. (2014) PMID: 24676634
263	CAKUT	c.448del	p.Thr150Argfs*9	Frameshift	No	6	KF (6)	Bower et al. (2012) PMID: 22213154
264	RCS	c.451delC	p.Pro151Argfs*6	Frameshift	Yes	8	KF (8)	Yang et al. (2021) PMID: 34696790

265	RCS	c.478_479insT	p.Ala160Valfs*21	Frameshift	Yes	14.7	KF (15.2)	Xiong et al. (2022) PMID: 35444690
266	FSGS	c.491C>A	p.Thr164Asn	Missense	No	8	KF (NA)	Barua et al. (2014) PMID: 24676634
267	CAKUT	c.497-2A>G	-	Splicing	No	2	KF (7)	Rossanti et al. (2020) PMID: 32203253
268	CAKUT	c.529G>A	p.Ala177Thr	Missense	NA	0.25	NA	Yang et al. (2021) PMID: 34696790
269	RCS	c.535_546delinsT	p.Asn179Trpfs*17	Frameshift	Yes	0.1	CKD G2 (5.9)	This study
270	CAKUT	c.543delA	p.Pro181Profs*92	Frameshift	No	11	KF (11)	Yang et al. (2021) PMID: 34696790
271	RCS	c.561del	p.Asn188Metfs*40	Frameshift	Yes	1.5	KF (15)	Sanyanusin et al. (1996) PMID: 7795640
272	RCS	c.561del	p.Asn188Metfs*40	Frameshift	Yes	NA	CKD G2 (10)	Sanyanusin et al. (1996) PMID: 7795640
273	RCS	c.561del	p.Asn188Metfs*40	Frameshift	Yes	Neonatal	KF (5)	Sanyanusin et al. (1996) PMID: 7795640
274	RCS	c.561del	p.Asn188Metfs*40	Frameshift	Yes	Childhood	CKD G3a (35)	Sanyanusin et al. (1996) PMID: 7795640
275	FSGS	c.565G>A	p.Gly189Arg	Missense	No	17	KF (40)	Barua et al. (2014) PMID: 24676634
276	CKD	c.565G>A	p.Gly189Arg	Missense	No	NA	KF (58)	Barua et al. (2014) PMID: 24676634
277	FSGS	c.565G>A	p.Gly189Arg	Missense	No	NA	Normal	Barua et al. (2014) PMID: 24676634
278	RCS	c.567_568dup	p.Ile190Argfs*39	Frameshift	Yes	1	NA	Megaw et al. (2013) PMID: 23686327
279	RCS	c.567_568dup	p.Ile190Argfs*39	Frameshift	Yes	NA	KF (NA)	Megaw et al. (2013) PMID: 23686327
280	CKD	c.567_568dup	p.Ile190Argfs*39	Frameshift	NA	NA	KF (17)	Megaw et al. (2013) PMID: 23686327
281	CKD	c.567_568dup	p.Ile190Argfs*39	Frameshift	NA	NA	KF (29)	Megaw et al. (2013) PMID: 23686327
282	CKD	c.567_568dup	p.Ile190Argfs*39	Frameshift	NA	NA	KF (28)	Megaw et al. (2013) PMID: 23686327
283	RCS	c.627delG	p.Glu209Glufs*65	Frameshift	Yes	2.2	KF (6)	Yang et al. (2021) PMID: 34696790
284	CAKUT	c.685C>T	p.Arg229*	Nonsense	NA	2.8	KF (6)	Yang et al. (2021) PMID: 34696790
285	CAKUT	c.686-1G>T	-	Splicing	No	9.1	CKD G2 (12.9)	This study
286	RCS	c.701C>G	p.Ser234*	Nonsense	Yes	NA	NA	Bower et al. (2012) PMID: 22213154
287	CAKUT	c.752T>A	p.Leu251*	Nonsense	No	NA	NA	Nicolaou et al. (2016) PMID: 26489027
288	RCS	c.754C>T	p.Arg252*	Nonsense	Yes	NA	CKD G3 (70)	Porteous et al. (2000) PMID: 10587573
289	RCS	c.754C>T	p.Arg252*	Nonsense	Yes	NA	KF (35)	Porteous et al. (2000) PMID: 10587573
290	RCS	c.754C>T	p.Arg252*	Nonsense	Yes	NA	CKD G3 (39)	Porteous et al. (2000) PMID: 10587573
291	RCS	c.754C>T	p.Arg252*	Nonsense	Yes	NA	KF (21)	Porteous et al. (2000) PMID: 10587573

292	RCS	c.754C>T	p.Arg252*	Nonsense	Yes	NA	Normal	Porteous et al. (2000) PMID: 10587573
293	RCS	c.754C>T	p.Arg252*	Nonsense	Yes	NA	KF (6)	Porteous et al. (2000) PMID: 10587573
294	CAKUT	c.754C>T	p.Arg252*	Nonsense	No	NA	KF (6)	Bower et al. (2012) PMID: 22213154
295	CAKUT	c.754C>T	p.Arg252*	Nonsense	NA	8	CKD G4 (8.8)	Xiong et al. (2022) PMID: 35444690
296	RCS	c.754C>T	p.Arg252*	Nonsense	Yes	3	CKD G3 (18.8)	This study
297	RCS	c.754C>T	p.Arg252*	Nonsense	Yes	0.33	CKD G2 (13)	This study
298	RCS	c.772C>T	p.258Gln*	Nonsense	Yes	NA	KF (NA)	Bower et al. (2012) PMID: 22213154
299	RCS	c.832C>T	p.Gln278*	Nonsense	Yes	14	KF (14)	Rossanti et al. (2020) PMID: 32203253
300	CAKUT	c.832C>T	p.Gln278*	Nonsense	No	6.1	CKD G3b (11.6)	This study
301	RCS	c.835del	p.Ala279Hisfs*18	Frameshift	Yes	NA	KF (13)	Bower et al. (2012) PMID: 22213154
302	RCS	c.836_c.840del AAGTC	p.Glu-279Glufs*11	Frameshift	Yes	11	KF (11)	Yang et al. (2021) PMID: 34696790
303	RCS	c.860delA	p.Gln287Argfs*10	Frameshift	Yes	10.1	CKD G3a (10.9)	This study
304	RCS	c.861+1G>A	-	Splicing	Yes	NA	NA	Bower et al. (2012) PMID: 22213154
305	CAKUT	c.861+1G>A	-	Splicing	NA	NA	KF (26)	Bower et al. (2012) PMID: 22213154
306	RCS	c.861+2T>C	-	Splicing	Yes	NA	KF (17)	Martinovic-Bouriel et al.(2010) PMID: 20358591
307	RCS	c.861+2T>C	-	Splicing	Yes	NA	KF (29)	Martinovic-Bouriel et al.(2010) PMID: 20358591
308	Nephrosis	c.862-1G>A	-	Splicing	No	NA	KF (27)	Vivante et al. (2019) PMID: 31001663
309	Nephrosis	c.862-1G>A	-	Splicing	No	NA	Normal	Vivante et al. (2019) PMID: 31001663
310	CAKUT	c.884C>T	p.Ala295Val	Missense	NA	NA	NA	Barua et al. (2014) PMID: 24676634
311	CAKUT	c.887T>C	p.Leu296Pro	Missense	NA	NA	NA	Barua et al. (2014) PMID: 24676634
312	RCS	c.890delC	p.Thr297fs	Frameshift	Yes	NA	KF (50)	Ciccone et al. (2024) abstract
313	RCS	c.890delC	p.Thr297fs	Frameshift	Yes	NA	CKD	Ciccone et al. (2024) abstract
314	CAKUT	c.892C>T	p.Pro298Ser	Missense	NA	NA	NA	Barua et al. (2014) PMID: 24676634
315	RCS	c.894delTinsGC	p.Gly299Argfs*3	Frameshift	Yes	Prenatal	CKD	Bower et al. (2012) PMID: 22213154
316	Nephrosis	c.906C>A	p.Tyr302*	Nonsense	NA	5	KF (13)	Yang et al. (2021) PMID: 34696790
317	Nephrosis	c.938C>T	p.Pro313Leu	Missense	No	14	Normal (NA)	Yang et al. (2021) PMID: 34696790

318	RCS	c.975C>A	p.Tyr326*	Nonsense	Yes	NA	NA	Bower et al. (2012) PMID: 22213154
319	CAKUT	c.985A>G	p.Thr329Ala	Missense	NA	NA	NA	Barua et al. (2014) PMID: 24676634
320	Ocular phenotype	c.988G>A	p.Gly330Ser	Missense	Yes	15	Normal (15)	Galvez-Ruiz et al. (2017) PMID: 29339962
321	RCS	c.1023C>A	p.Tyr341*	Nonsense	Yes	NA	CKD G3a (28)	Okumura et al. (2015) PMID: 26571382
322	CAKUT	c.1052G>T	p.Gly351Val	Missense	No	0.1	CKD G3b (1.6)	This study
323	Nephrosis	c.1127A>C	p.Gln376Pro	Missense	NA	37	KF (48)	Yang et al. (2021) PMID: 34696790
324	Nephrosis	c.1127A>C	p.Gln376Pro	Missense	NA	27	CKD G2 (NA)	Yang et al. (2021) PMID: 34696790
325	Nephrosis	c.1127A>C	p.Gln376Pro	Missense	NA	35	KF (NA)	Yang et al. (2021) PMID: 34696790
326	RCS	c.1160G>A	p.Ser387Asn	Missense	Yes	NA	NA	Bower et al. (2012) PMID: 22213154
327	FSGS	c.1178G>C	pArg393Pro	Missense	No	NA	NA	Mansilla et al. (2021) PMID: 31738409
328	CAKUT	Genomic deletions	Gene deletion	Large deletion	No	3	KF (9.5)	Xiong et al. (2022) PMID: 35444690

CAKUT, congenital anomalies of the kidney and urinary tract; RCS, renal coloboma syndrome; FSGS, Focal segmental glomerulosclerosis; pLOF, predicted loss of function; KF, kidney failure. *Stages of chronic kidney disease (CKD G2–5) are defined as follows: G1, eGFR ≥ 90; G2, eGFR 60–89; G3a, eGFR 45–59; G3b, eGFR 30–44; G4, eGFR 15–29; G5, eGFR <15 or treated by dialysis [eGFR, estimated glomerular filtration rate in mL/min/1.73 m^2, is calculated using the Schwartz formula.]

Supplementary Fig. S1. Synopsis of the clinical course in patients with *PAX2* pathogenic variants

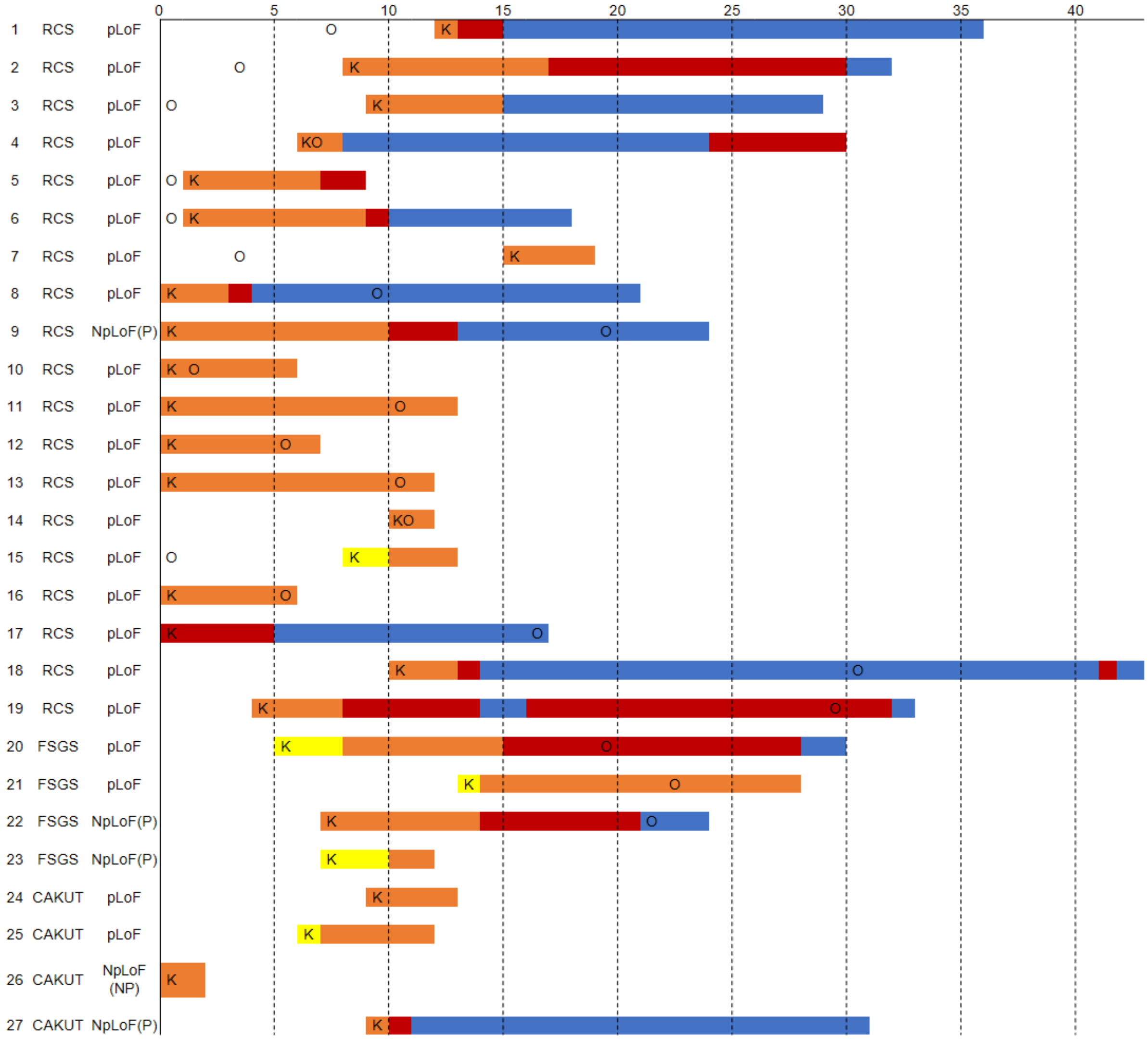

The first and second columns indicate patient ID and clinical diagnosis. The third column describes the type and site of *PAX2* mutations. The symbols denote the time of initial kidney and ocular manifestations. The yellow bars denote the time before the development of CKD, the orange bars denote the period with CKD, the red bars show dialysis periods, and the blue bars show transplantation periods.

RCS, renal coloboma syndrome; FSGS, focal segmental glomerulosclerosis; CAKUT, isolated congenital anomalies of the kidney and urinary tract; pLoF, predicted loss of function; NpLoF, non-predicted loss of function; P, paired domain; NP, non-paired domain; K, kidney; O, ocular; CKD, chronic kidney disease

References

1. Cheong HI, Cho HY, Kim JH, Yu YS, Ha IS, Choi Y. A clinico-genetic study of renal coloboma syndrome in children. *Pediatr Nephrol.* 2007;22(9): 1283-1289.
2. Ahn YH, Lee C, Kim NKD, et al. Targeted Exome Sequencing Provided Comprehensive Genetic Diagnosis of Congenital Anomalies of the Kidney and Urinary Tract. *J Clin Med.* 2020;9(3): 751.
3. Park E, Lee C, Kim NKD, et al. Genetic Study in Korean Pediatric Patients with Steroid-Resistant Nephrotic Syndrome or Focal Segmental Glomerulosclerosis. *J Clin Med.* 2020;9(6): 2013.
4. Jung J, Lee JH, Park YS, et al. Ultra-rare renal diseases diagnosed with whole-exome sequencing: Utility in diagnosis and management. *BMC Med Genomics.* 2021;14(1): 177.
